# Co-Evolution of Sink and Source in the Recent Breeding History of Winter Wheat in Germany

**DOI:** 10.3389/fpls.2019.01771

**Published:** 2020-02-07

**Authors:** Carolin Lichthardt, Tsu-Wei Chen, Andreas Stahl, Hartmut Stützel

**Affiliations:** ^1^Vegetable Systems Modelling Section, Institute of Horticultural Production Systems, University of Hannover, Hannover, Germany; ^2^Department of Plant Breeding, IFZ Research Centre for Biosystems, Land Use and Nutrition, Justus Liebig University, Giessen, Germany

**Keywords:** winter wheat, breeding progress, complementary crosses, sink and source, green canopy duration

## Abstract

Optimizing the interplay between sinks and sources is of crucial importance for breeding progress in winter wheat. However, the physiological limitations of yield from source (e.g. green canopy duration, GCD) and sink (e.g. grain number) are still unclear. Furthermore, there is little information on how the source traits have been modified during the breeding history of winter wheat. This study analyzed the breeding progress of sink and source components and their relationships to yield components. Field trials were conducted over three years with 220 cultivars representing the German breeding history of the past five decades. In addition, genetic associations of QTL for the traits were assessed with genome-wide association studies. Breeding progress mainly resulted from an increase in grain numbers per spike, a sink component, whose variations were largely explained by the photosynthetic activity around anthesis, a source component. Surprisingly, despite significant breeding progress in GCD and other source components, they showed no direct influence on thousand grain weights, indicating that grain filling was not limited by the source strength. Our results suggest that, 1) the potential longevity of the green canopy is predetermined at the time point that the number of grains is fixed; 2) a co-evolution of source and sink strength during the breeding history contribute to the yield formation of the modern cultivars. For future breeding we suggest to choose parental lines with high grain numbers per spike on the sink side, and high photosynthetic activity around anthesis and canopy duration on the source side, and to place emphasis on these traits throughout selection.

## Introduction

The final grain yield of winter wheat is the result of the number of grains and the grain weight which are determined by the primary yield components: spike number per unit area, grain number per spike, and thousand grain weight (TGW) (Fischer, 2011). The formation of different yield components can be interpreted as the result of the interplay between sinks and sources. Sink size of the developing yield organs is determined by the number of spikes per unit area, grains per spike, and a specific sink size per grain (resulting in TGW). Source size is related to the production of photo-assimilates, namely the size, the photosynthetic capacity, and the duration of the leaf area, which drive spike development and grain filling (Jagadish et al., 2015).

The interplay between sinks and sources has an impact on yield formation which becomes apparent from double ridge stage (first spikelet ridges visible) to the end of grain filling. In the very early phase, from double ridge to terminal spikelet stages, a source limitation may result in a reduced spikelet number (Guo et al., 2018). Around anthesis, spikelet fertility is affected and the physiological process of floret abortion could be activated, thereby reducing the grain number. The physiological factors relevant for source limitation at anthesis are 1) the canopy leaf area, which maximizes light interception, and 2) photosynthetic capacity per leaf area which maximizes the utilization of light energy for production of plant mass. It has been shown that limitation of source strength (e.g. by shading of leaves around anthesis) reduces grain number per spike (Wang et al., 2003). Furthermore, the crop growth rate around anthesis could also be associated with grain number (Bancal, 2008; Guo et al., 2018). This indicates a negative effect of source limitation around anthesis on the formation of the sink size, namely grain number. After the seed number is determined, a reduction in source limitation can be achieved physiologically by extending the canopy longevity, or the capacity to stay green. Source limitation during the grain filling phase (after anthesis) can reduce the TGW (Foulkes et al., 2009; Guo and Schnurbusch, 2015; Liang et al., 2018), indicating a negative effect of the source limitation after anthesis on the sink size, namely grain weight.

An ongoing debate is whether grain yield is sink or source limited. Complementary crosses between genotypes with high sink capacity and those with high source capacity resulted in progeny with substantial yield improvement (Reynolds et al., 2017), suggesting the co-limitation of source and sink on yield. It has been suggested that the selection of crossing partners based on physiological traits is a promising strategy to achieve a higher crop productivity via breeding, which is not least facilitated by the increasingly automated phenotyping techniques (Foulkes et al., 2009; Reynolds and Langridge, 2016; Reynolds et al., 2017; Furbank et al., 2019). A precise description of the sink and source characteristics of possible genetic resources for future winter wheat varieties is, together with the application of genomic tools, a purposive strategy to promote the genetic gain via strategic complementary crosses. Functional interdependencies of physiological traits often depend on pleiotropic relationships or interactions of relevant genetic loci. Therefore, patterns of co-evolution during breeding history between interacting sinks and sources can be assumed (Schulthess et al., 2017; Alonso et al., 2018). In order to meet the projected demand for food and the challenges of climate change (Reynolds et al., 2009; Ray et al., 2013), possible linchpins to further increase the yield potential of new cultivars can be found by investigating physiological and genetic interdependencies between yield, yield components, and their links to source and sink components.

The co-limitation of sink and source implies that breeding progress of them should be achieved parallelly. However, the concrete interactions of source characteristics with the sink traits and thereby its role in breeding progress of winter wheat is unclear, especially for the capacity of the canopy to stay green (Jagadish et al., 2015). Since stay-green prolongs the time for carbon assimilation and increases the source for grain filling (Lawlor and Paul, 2014), it can be hypothesized that, under source limited conditions, breeding progress in stay-green trait is linked to the breeding progress in TGW. The beneficial effect of stay-green traits on grain yield, especially TGW, has been demonstrated and summarized by several research groups (Wu et al., 2012; Gregersen et al., 2013; Xie et al., 2016). Additionally, previous studies have shown the link between increased leaf area index (LAI) or extended longevity of the leaf area and yield increase in modern varieties (Tian et al., 2011; Sanchez-Garcia et al., 2015). However, these studies did not elaborate, whether grain number or TGW are affected by the delayed senescence, so that the functional interface between sink and source remains unclear. Additionally, especially for the central European wheat cultivars, the interdependencies of sink and source characteristics have to our knowledge not yet been tested in greater detail. Interestingly, there is evidence of a non-causal association between source and sink activities during grain filling and a possible link between a delayed senescence and sink size, namely grain number (Yin et al., 2009; Liang et al., 2018). In summary, one would at first glance assume a direct effect of the stay-green trait on thousand grain weight but there could also be a link from grain number to an extended availability of photosynthetic active materials.

The present study aims to decipher the source-sink interdependencies between the yield components during grain filling and to evaluate the contributions of German breeding progress on the sink and source characteristics after anthesis. We used 220 cultivars, 174 of which were released in Germany between 1966 and 2013, representing the breeding history of the last five decades. All cultivars were grown over three consecutive seasons (2014–2017) to study the breeding progress of the source strength (leaf area index, relative chlorophyll content, and canopy longevity) and the sink strength (spike number, grain per spike, and grain weight). We hypothesized that high yield of modern wheat cultivars is realized by both a higher grain number and higher TGW with the first being associated with the photosynthetic capacity and leaf area at flowering, whereas the latter is linked to the increase in canopy longevity to assure the source for grain filling. Furthermore, a genome wide association study (GWAS) was conducted to identify the significant quantitative trait loci (QTL) to facilitate marker-assisted selection.

## Materials and Methods

### Plant Materials

Breeding history of German winter wheat was represented by a collection of 174 wheat cultivars released between 1966 and 2013. These cultivars, including 5 hybrids and 169 pure lines, are recommended for conventional production and represent all baking quality classes “E,” “A,” “B,” and “C” (very high to very low baking quality). They were selected based on their economical importance in Germany. Each decade in the breeding history was represented by more than 20 cultivars and 66% of the collection were released in the last two decades (**Figure S1**). Additionally, 46 diverse accessions obtained from the German seed bank (https://gbis.ipk-gatersleben.de) were included to enlarge the genetic diversity and improve the reliability of the genome-wide association study (see later section). In total, 220 cultivars were used in this study and the complete list of cultivar names, year, and country of registration, breeder, quality classification, and the assignment to the subsets is provided in **Table S1**. All cultivars of the breeding history subset of this study were included in the main experiment of the study described by Voss-Fels et al. (2019a).

### Experimental Design and Growing Conditions

Field trials were conducted in three seasons (2014–2015, 2015–2016, and 2016–2017) at the research station in Ruthe near Hannover (52°14’44.1"N 9°49’03.4"E, clayey silt soil type). All 220 cultivars were sown in plots with 330 viable seeds m^2^ and in 15 rows in 2 m plot width (13.33 cm row spacing). The plot sizes were 12 m², 10 m², and 9.4 m² in the three consecutive years, respectively. Plots were arranged in a randomized block design with two replications and cultivars were randomized within four sub-groups according to the flowering time and plant height (early and short; early and tall; late and short; late and tall) based on previous knowledge (tall = >100cm height). The plots were treated according to standard agrochemical application in intensive wheat production in Germany (**Table S2**). Due to the field design, applications were conducted on all plots when most cultivars reached the relevant stage for application and they were all treated once. Mineral nitrogen (N) fertilizer was supplied in three applications with a target value of 220 kg N/ha including the soil mineral nitrogen (N_min_) measured at the beginning of the growing season in the root zone (Wehrmann and Scharpf, 1979). In each season, growth regulators were applied once at stem elongation stage and fungicides were applied at stem elongation, flag leaf appearance, and beginning of flowering. The treatment of growth regulator followed the recommendation of regional advisers with expertise in winter wheat production to be in step of the actual best-practice. Weed and insect control were applied according to the requirements. A summary of the crop protection is provided in **Table S2** and the weather conditions during the experimental periods are summarized in **Figure S2**.

### Yield Measurements

Shortly before harvesting the plots, a sample of one row (50 cm in length) was cut to determine the harvest index (HI) by the ratio of grain yield (g m^-2^) to total dry biomass (g m^-2^). Numbers of spikes and TGW (g) of these samples were used to determine spikes per m² and grains per spike and m². Plot grain yield and TGW were determined by harvesting the complete plots with a combine harvester. Plot biomass was calculated by dividing the grain yield by the harvest index.

### Physiological Measurements

Heading date [BBCH59, (Witzenberger et al., 1989)] was recorded for all cultivars in one replication per year. Additionally, the hard-dough (BBCH87) was recorded in one replication each year. Because the investigation is a time-consuming process, the hard-dough was only recorded for a subset of 20 cultivars, which were selected to represent the variation in maturity. Due to the fungicide treatment, leaf and ear diseases, including powdery mildew, rust, *Septoria spp*., and *Fusarium*, were successfully controlled. Therefore, all cultivars were close to 100% green at the heading stage. After heading, the declining fraction of green leaf area (%) was visually scored every one to two weeks. Around the heading date (approx. 230 days after sowing), the leaf area index was maximal and was measured by a plant canopy analyzer (LAI-2200C, Li-COR, Lincoln, Nebraska USA).

To quantify the dynamics in the senescence pattern, a logistic power function with two parameters was used to describe the relationships between the fraction of green leaf area (y, %) and the thermal time (TT, °Cd):

(1) $$\text{y }=\;\frac{1}{1+{\left(\frac{\text{TT}}{{\text{GLA}}_{50}}\right)}^{\text{s}}}$$

GLA_50_ is the temperature sum (°Cd), at which the green leaf area drops to 50% and s describes the steepness of the curve (**Figure S3**). Temperature sum is defined as the cumulative sum of the daily mean temperatures, starting from the day of sowing with 0°C as base temperature. Green canopy duration (GCD, °Cd) was defined as the difference between GLA_50_ and the thermal time at heading date (TT_heading_):

(2) $$\text{GCD}={\text{ GLA}}_{50}-{\text{TT}}_{\text{heading}}$$

In addition, leaf area duration (LAD) was defined as the product of the maximal leaf area index (LAI_max_) and duration of it, namely the integral of the logistic curve from heading to harvest:

(3) $$\text{LAD}=\int_{TT_{heading}}^{TT_{harvest}}\frac{1}{1+{\left(\frac{TT}{GLA_{50}}\right)}^{\text{s}}}\;\times {\text{ LAI}}_{\text{max}}$$

LAI_max_ is a measure for the canopy development until heading and a proxy of the biomass accumulation prior to grain filling. GCD is defined as the duration of the availability of photosynthetic leaves from heading to 50% leaf senescence (**Figure S3**). LAD is the integral of the green leaf area from heading to harvest weighted by the maximal canopy size (LAI_max_), in other words the product of the integrated canopy duration and the canopy size. The photosynthetic activity is difficult to assess in a large scale field experiment. Therefore, the SPAD values of the flag-leaf were measured as a proxy for the photosynthetic activity in 2016 and 2017. For each genotype and replication, five flag leaves were measured at the widest section around the heading dates of that genotype (±10 days).

### Statistical Analyses

The phenotypic data collected in the field experiment was evaluated with the following mixed model

(4) $$P_{ijkl}=\mu +\;c_{i}+\;y_{j}+\;cy_{ij}+\;YR_{jk}+\;YRG_{jkl}+\;e_{ijkl}$$

where *P_ijkl_* was the phenotypic observation of the *i*^th^ cultivar (*i = cultivar number 1 – 220, factorial*) in the *j*^th^year (*j = 2015, 2016 and 2017, factorial*) in the *k*^th^ complete replication and the *l*^th^ incomplete sub-group (*l = early and short; early and long late and short; late and short*). Fixed factors are indicated by lowercase letters, capital letters indicate random effects. The observation was dissected into the general mean, *μ*, the genetic effect of the *i*th cultivar *c_i_*, the effect of the *j*th season *y_j_*, the interaction of the cultivar and the season *cy_ij_*, the interaction of the sub-group, the replication and the season *GRY_jkl_*, the interaction of the replication and the season *RY_jl_* and the residual *e_ijkl_*. The model was fit to the data with the *lmer* function of the *lme4* package in the R environment (Bates et al., 2015; R Core Team, 2017). Significant differences between cultivars, seasons, and the interactions were examined with the *anova* function. The best linear unbiased estimators (BLUE) for each cultivar and cultivar within each year were calculated using the *lsmean* function from the *lsmeans* package (Lenth, 2016). Analyses of the cultivar’s performances within each year and across the years was performed based on the estimated BLUEs and were also used the phenotypic values in the marker-trait associations in the genome-wide association study.

Broad-sense heritability *H^2^* of the physiological and yield components within the breeding history-subset was calculated over *n* environments and *r* replications according to the formula:

(5) $$H^{2}=\;\frac{\sigma_{C}^{2}}{\left(\sigma_{C}^{2}+\;\frac{\sigma_{CY}^{2}}{n}+\;\frac{\sigma_{e}^{2}}{nr}\right)\;}$$

where $\sigma_{C}^{2},\;\sigma_{CY}^{2}$ and $\sigma_{e}^{2}$ are the genetic variance component, the interaction variance component between genotype and environment, and the residual variance component (equation [4]), respectively. To estimate the variance components, the model in equation (4) was set as completely random.

### Quantification of the Breeding Progress

The population of 174 cultivars was used to quantify the breeding progress in winter wheat. The *absolute breeding progress* (increase per year) was the slope of the linear regression line between the year of release and the parameter of interest. The linear regressions were calculated based on sliding window means, where the window for mean calculation is moving on the scale of the year of release with a constant window size of 10 cultivars with cultivars ordered by the year of release. Means and standard deviation of the parameters in each window were calculated. Using the linear regression equation of the absolute breeding progress, the *relative four-decades breeding progress* (%) of the parameters was described by the ratio between the trait values of 2010 and 1970. It is an estimate for the superiority of the modern cultivar in percent (in the following referred to as *relative breeding progress*). The breeding progress was investigated for each experimental year separately (BLUE values per cultivar and year) and on average (BLUE values per cultivar).

### Genome-Wide Association Study

Genome wide association study (GWAS) was performed to identify marker-trait-associations of the single nucleotide polymorphisms (SNPs) associated with the parameters which were putatively improved with breeding. Leaf DNA samples of the total collection of 220 accessions were genotyped with the 135K Affymetrix TGWEXCAP Array carrying a total of 136,780 SNP markers (TraitGenetics, Gatersleben, Germany; see **Supplementary Data Sheet 2** of this paper and of Voss-Fels et al., 2019a). The complete set of markers was applied to detect clusters of genetically related individuals within the R package *adegenet* (Jombart, 2008). A discriminant analysis of principal components (DAPC) was performed. *Via* a k-means clustering of initial calculated principle components, five groups were identified. These groups were then implemented as covariates in the mixed model which was applied to estimate the genome wide association.

To anchor the SNP markers to physical positions, 136,780 SNP probes were aligned to the *T. aestivum* genome [IWGSC release iwgsc_refseqv1.0 assembly soft-masked version (IWGSC, 2018)] using BLASTN 2.2.31 (Camacho et al., 2009). Markers were excluded if their SNP probe sequence could not be aligned with high stringency to a unique physical position on the reference sequence (E-value ≤ 10^-5^). The results were filtered with the following criteria 1) uniquely mapped, 2) no gap, and 3) minimum 1 base mismatch, to obtain a total of 92,464 anchored SNP markers. After quality control by filtering monomorphic markers with >10% missing values or a minor allele frequency <5%, a selection of 45,370 high-quality, polymorphic SNPs remained in the data set for further analyses. On average 2,130 markers per chromosome were applied for the genome-wide scan for marker-trait assassinations. The size of the area on the chromosome covered with markers ranged from 473 Mbp (chromosome 6D) to 829 Mbp (chromosome 3B) and hence the marker density lied between one marker per 1.6 Mbp (chromosome 4D) and one marker per 0.2 Mpb (chromosome 5B). The minor allele frequency ranged from 0.19 (chromosome 3D) to 0.28 (chromosome 6A). The allelic associations were calculated for genotypic trait values (BLUE values) of yield, HI, biomass, grains per spike, SPAD, LAImax, and GCD with the polygenic function in the R package genABEL by implementing the population structure and the genome wide kinship matrix (Aulchenko et al., 2007). The Bonferroni method (*p* < 0.05) and the false discovery rate (FDR 10%) were considered as thresholds for significant marker-trait-associations.

## Results

### Environmental Effects on Yield Component Traits and Source Characteristics

**Figure 1:**
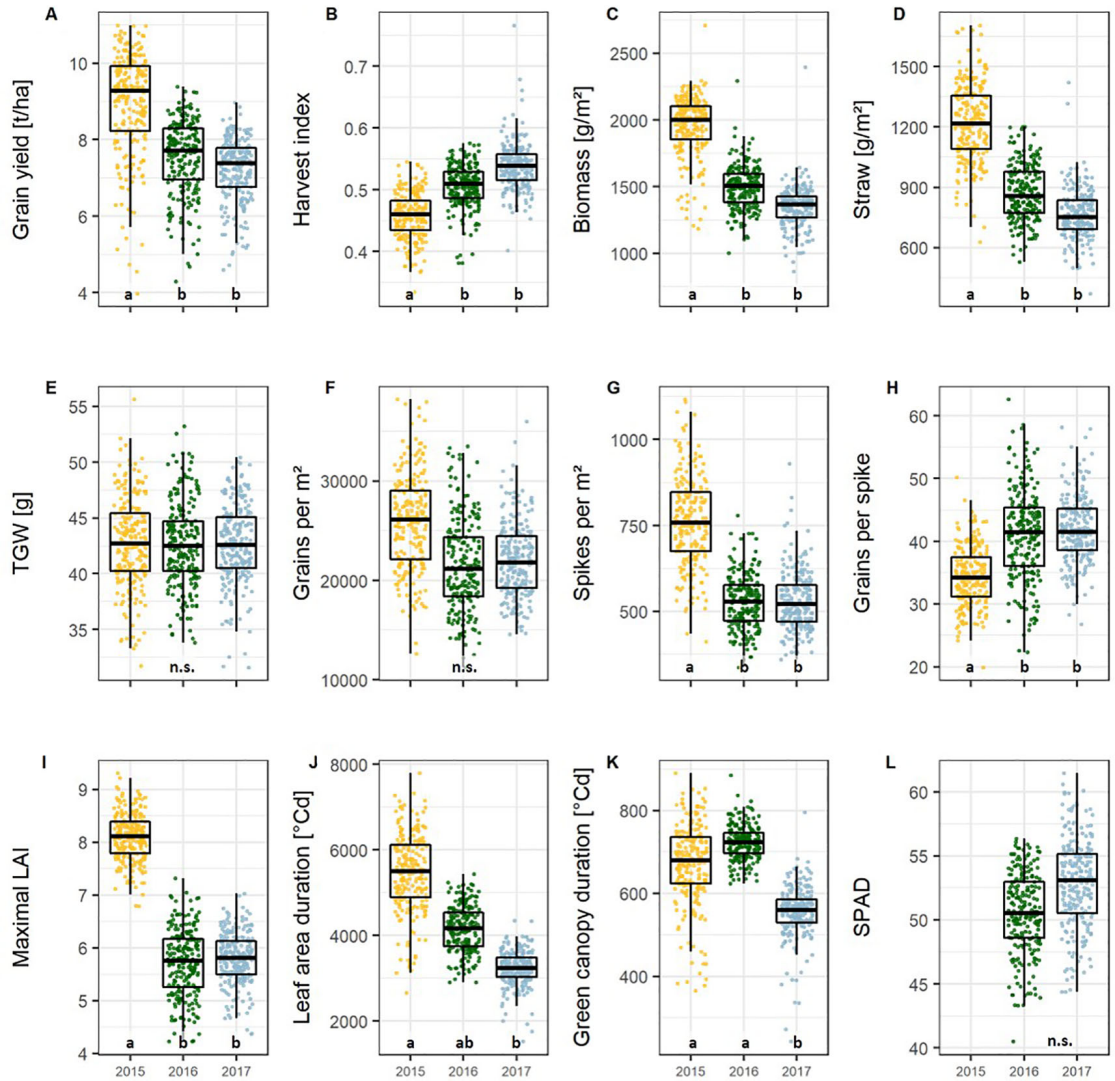
Boxplots of the **(A)** yield, **(B–H)** yield components, and, and **(I–L)** green canopy parameters presenting the mean values per cultivar and season for the 174 cultivars representing the breeding history. Different lower-case letters within the plots indicate significant differences between seasons. Abbreviations are listed in **Table 1**.

All traits showed significant differences between the 174 wheat cultivars representing the breeding history (**Table S3**, *p <* 0.05). Between the growing seasons all parameter values differed significantly except TGW (**Figure 1E**), grains per m², and SPAD (**Figure 1L**). The interaction between growing season and cultivar was significant for all traits except for biomass, HI, spikes per m², and grains per m².

In 2015 the mean grain yield of all cultivars was around 17% higher than in 2016 and 2017, but the HI was lower in 2015 compared to 2016 and 2017 (**Figures 1A, B**). This was probably due to the high nitrogen availability during the early vegetative growth in 2015 (**Table S4**), the high ratio of daily radiation to mean temperature (data not shown), and an overall lower mean temperature (**Table S5**), which significantly enhanced the vegetative growth indicated by 27% more total biomass (**Figure 1C**) and 33% more straw (**Figure 1D**). In parallel, spike number, determined by the physiological processes related to tillering and tiller reduction during the vegetative development, was about 30% higher in 2015 (**Figure 1G**). However, the higher spike number in 2015 was accompanied by a 17% lower number of grains per spike (**Figure 1H**). The total amount of grains per unit area, which is the product of the number of spikes and number of grains per spike, remained only slightly higher in 2015 (not significant) than in the other seasons (**Figure 1F**). This indicates a higher robustness of this sink trait, or vice versa a higher plasticity of spike number and grains per spike with respect to environmental conditions.

The maximal leaf area index (LAI_max_), SPAD, and the canopy longevity parameters, including green canopy duration (GCD, eqn. 2) and leaf area duration (LAD, eqn. 3), were taken as the parameters describing source capacity. All parameters showed significant cultivar and cultivar by year effects, and, except for SPAD, significant differences between years (**Table S3**). For the source parameters, the heritabilities were lower than that for yield and grains per spike (0.50, 0.66, 0.51, and 0.57 for LAI_max_, SPAD, LAD, and GCD, respectively, **Table S6**), indicating a high environmental variance of the traits.

Conditions for vegetative growth were more favorable in 2015 resulting in a 28% higher maximum LAI than in the other two seasons (**Figure 1I**). In 2017, the canopy longevity parameters LAD and GCD showed significantly lower values due to high temperatures in the later generative phase (**Figures 1J, 1K**, and **S2**). High LAD values in 2015 can be attributed to the high maximal LAI values.

### The Main Factor Relevant for Yield Formation, Grains Per Spike, Is Determined by the Photosynthetic Activity Around Anthesis and Affects the Canopy Longevity

**Figure 2:**
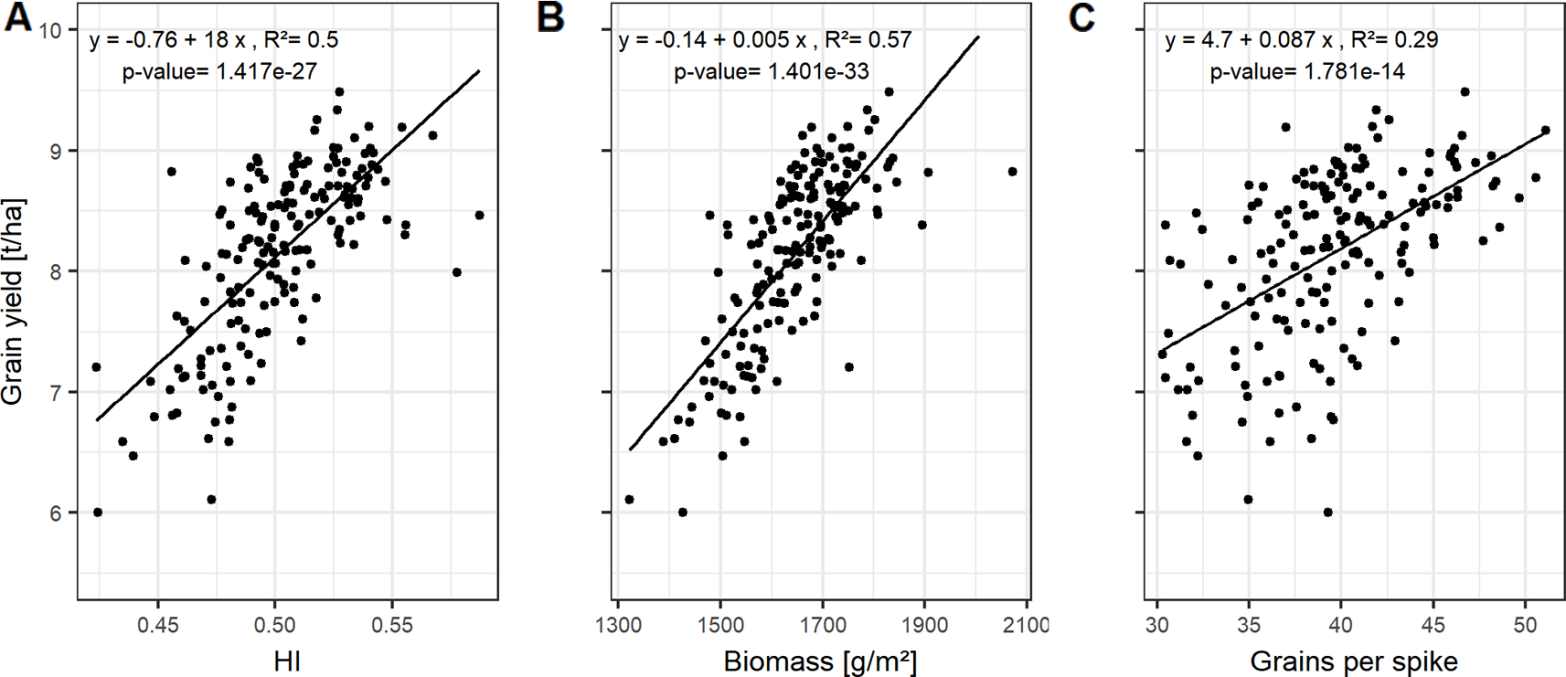
Relationships between grain yield and **(A)** harvest index (HI); **(B)** biomass; and **(C)** grains per spike. Each point represents the mean values of a cultivar in three growing seasons. In total, 174 cultivars representing the breeding history were used.

HI and biomass explain variations in grain yield with high accuracies, indicated by the high correlation values on average (**Figures 2A, B**) and for each of the growing seasons (**Figure S4**). The interdependencies of the source characteristics (LAI_max_, SPAD, LAD, and GCD) and these yield parameters indicate that both, the photosynthetic activity (SPAD) and the longevity of the canopy (GCD) are of importance for yield formation.

However, a positive relationship between wheat leaf photosynthesis and leaf chlorophyll content has been widely observed (Wu et al., 2009) and the leaf chlorophyll content, in turn, is closely associated with non-destructive measurements with the hand-held digital chlorophyll meter SPAD (SPAD 502, Minolta, Japan) (Bannari et al., 2007).

HI, the ratio of grain yield to total biomass, correlates mainly with the GCD, whereas the total biomass can be not only associated to the size of photosynthetic leaf material, but also by activity (SPAD-values) of assimilate production around anthesis (**Figure S4**).

**Figure 3:**
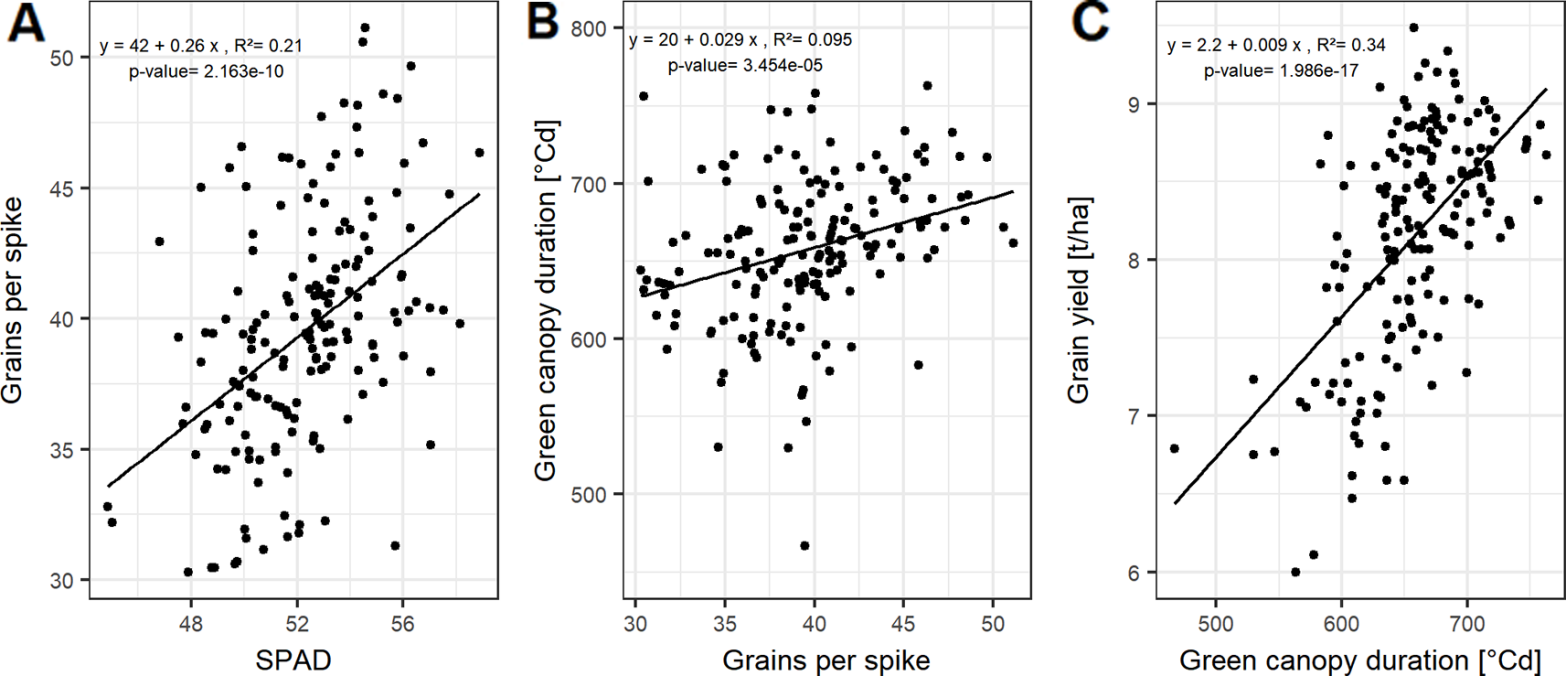
Relationships between **(A)** grains per spike and SPAD; **(B)** green canopy duration and grain per spike; and **(C)** grain yield and green canopy duration. Each point represents the mean values of a cultivar in three growing seasons. In total, 174 cultivars representing the breeding history were used.

Grain yield can be dissected into thousand grain weight and grain number per unit area, the latter being the product of grains per spike and spike number per unit area. However, the genotypic differences in grain yield could not be explained by the genotypic variation in spike number and TGW of the studied cultivars (**Figure S4**). In contrast, grains per spike explained the variation in grain yield with a Pearson coefficient of correlation (*r*) of up to 0.54 averaged over all seasons (**Figures S4** and **2C**). Linear relationships between the yield components and the source characteristics indicate that grain number per spike was mainly influenced by the photosynthetic capacity around anthesis. The SPAD values explained 21% of the variation of grain number per spike when averaged over the three seasons (**Figure 3A**).

For a subset of 20 cultivars, the grain filling duration (temperature sum of BBCH59 subtracted from temperature sum of BBCH87) showed on average a significant correlation with GCD with *R*² = 0.60 (**Figure S5**). This tight relationship indicates a physiological link between GCD and the grain filling duration. Grain number per spike was significantly related to GCD (**Figures 3B** and **S4**). The influence of grains per spike on grain yield is therefore in parts indirectly mediated by the canopy duration, which explains 34% of the variation in grain yield (**Figure 3C**). Surprisingly, insignificant correlation between GCD and TGW rejected our hypothesis and suggested that the influence of canopy duration and yield formation was already determined at the beginning of the grain filling phase. The source activity around anthesis affects the grain number which then decides on the durability of the source to fill the grains.

### Breeding Progress Was Most Pronounced in Grains Per Spike and Green Canopy Duration

**Figure 4:**
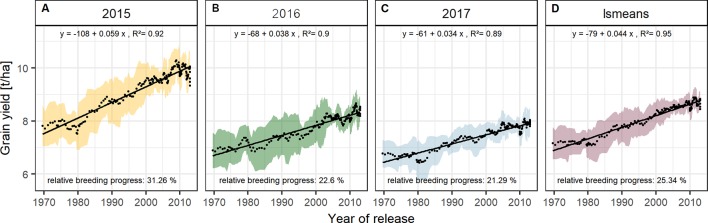
Sliding window plots showing breeding progress in grain yield per season **(A–C)** and on average **(D)**. Each dot represents a mean value of a group of 10 cultivars and the colored area represents their standard deviations. The slopes of the linear regression lines (black line) are referred to as absolute breeding progress and the relative breeding progress is the ratio between the values in 2010 and 1970.

The absolute breeding progress in yield between 1970 and 2010 was clearly linear and was nearly twice as high in 2015 than in 2016 and 2017 (**Figure 4**). On average, the grain yield in 2015 was 7.55 t/ha for the cultivars released in the 1970s, increased annually by 59 kg/ha and reached 9.85 t/ha for cultivars released in 2010, indicating a relative breeding progress of 31.3 % in grain yield between 1970 and 2010 (**Figure 4A**). The corresponding values for 2016 and 2017 were 22.6 % and 21.3 %, respectively (**Figures 4B, C**).

Grain yield can be expressed as the product of biomass and harvest index (HI). The relative breeding progress in yield from 1970 to 2010 may be considered as the result of changes in biomass and HI:

$$\;\frac{Yield_{2010}}{Yield_{1970}}\;=\;\frac{Biomass_{2010}}{Biomass_{1970}}\times \frac{HI_{2010}}{HI_{1970}}$$

**Figure 5:**
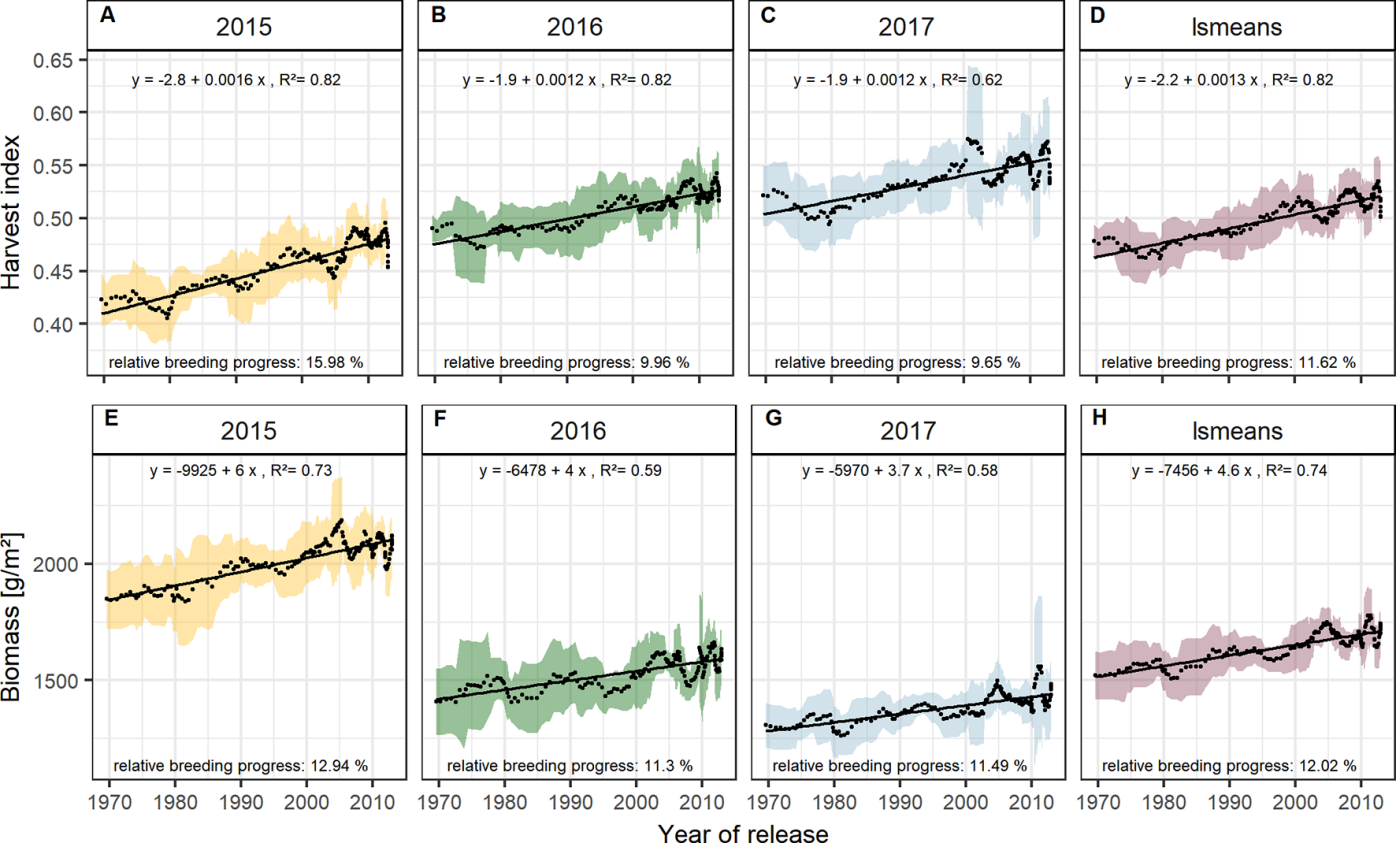
Sliding window plots showing breeding progress of harvest index **(A–D)** and biomass **(E–H)**. For detail, see the caption of **Figure 4**.

With approx. 12 % the relative breeding progress in biomass was similar in all three experimental years (**Figures 5E–H**), while the relative breeding progress in HI in 2015 was 15.98 %, about 6 % higher than the progress measured in 2016 and 2017 (**Figures 5A–D**). This indicates that, in comparison with 2016 and 2017, the larger differences in yield between old and new cultivars grown in 2015 were due to their differences in HI. This agrees with the fact that in 2015 the correlation of yield with HI was higher than with biomass. In contrast, biomass showed the highest correlation with yield among all yield components in 2016, 2017, and on average (**Figures 2** and **S4**).

**Figure 6:**
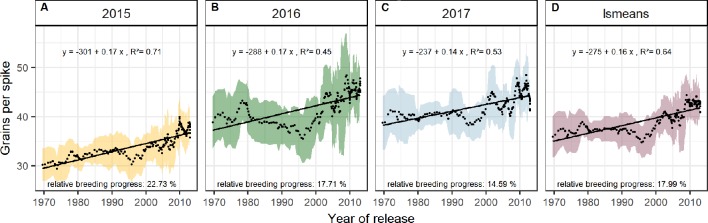
Sliding window plots showing breeding progress of grains per spike **(A–D)**. For detail, see the caption of **Figure 4**.

Dissecting the breeding progress into that of the yield components, it is remarkable that the relative breeding progress of TGW was generally low and only conspicuous in 2015, showing an increase of 10% from 1970 to 2010 (**Table 1**). Accordingly, the correlations between TGW and grain yield, HI and the year of release were also higher in 2015 (**Figure S4B**). A slightly higher relative breeding progress in 2015 was also observed for all tested source parameters. However, the progress of grain number was lower than in the other years and spikes per m² even showed a negative genetic trend in 2015, indicating that the modern cultivars developed 1.6 spikes per m² less than the old cultivars. Despite having a lower spike number in 2015, the modern cultivars still developed higher yields than the older cultivars due to a 22.7% increase in grains per spike (**Figure 6**). Among all yield components, grains per spike had the highest relevance for breeding progress. This was indicated by the highest correlations with the year of release within the seasons and on average (**Figure S4**) and high relative breeding progress (**Table 1**).

**Table 1:** Breeding progress of the main yield and stay green parameters dissected into the development within each growing season separately and calculated for all seasons together (overall).

	2015			2016			2017			Overall		
	absolute	*R*²	relative	absolute	*R*²	relative	absolute	*R*²	relative	absolute	*R*²	relative
**Grain yield**	0.06	0.92	31.26	0.04	0.90	22.60	0.03	0.89	21.29	0.04	0.95	25.34
**HI**	0.0016	0.82	15.98	0.0012	0.82	9.96	0.0012	0.62	9.65	0.0013	0.82	11.62
**Biomass**	5.98	0.73	12.94	4.01	0.59	11.30	3.68	0.58	11.49	4.55	0.74	12.02
**Straw**	−1.34	0.09	−4.12	0.77	0.04	3.59	n.s.			n.s.		
**TGW**	0.10	0.54	9.86	n.s.			0.06	0.43	5.50	0.05	0.32	4.83
**Grains/m²**	75.70	0.40	12.51	122.46	0.52	26.66	81.72	0.58	16.67	93.29	0.76	18.00
**Spikes/m²**	−1.49	0.16	−7.26	0.89	0.15	7.15	n.s.			n.s.		
**Grains/spike**	0.17	0.71	22.73	0.17	0.45	17.71	0.14	0.53	14.59	0.16	0.64	17.99
**LAImax**	n.s.			0.02	0.45	11.36	−0.01	0.27	−3.69	0.00	0.20	2.64
**LAD**	27.20	0.79	22.55	15.16	0.50	16.36	8.38	0.36	10.98	16.92	0.74	17.52
**GCD**	3.17	0.84	21.26	0.81	0.44	4.63	2.07	0.57	16.47	2.02	0.79	13.46
**SPAD**				0.07	0.50	5.77	0.11	0.59	8.42	0.09	0.62	7.11

Absolute and relative breeding progress was derived from sliding window means. Units for absolute breeding progresses are: grain yield: t ha^-1^; harvest index (HI): unitless; biomass: g m^-^²; straw: t ha^-1^; thousand grain weight (TGW): g; grain m^-^²; spikes m^-^²; grain spike^-1^; maximal leaf area index (LAI_max_): unitless; green canopy duration (GCD): °Cd; leaf area duration (LAD); and SPAD (unitless). Relative breeding progress is expressed in percent (%). n.s. indicates p < 0.05.

**Figure 7:**
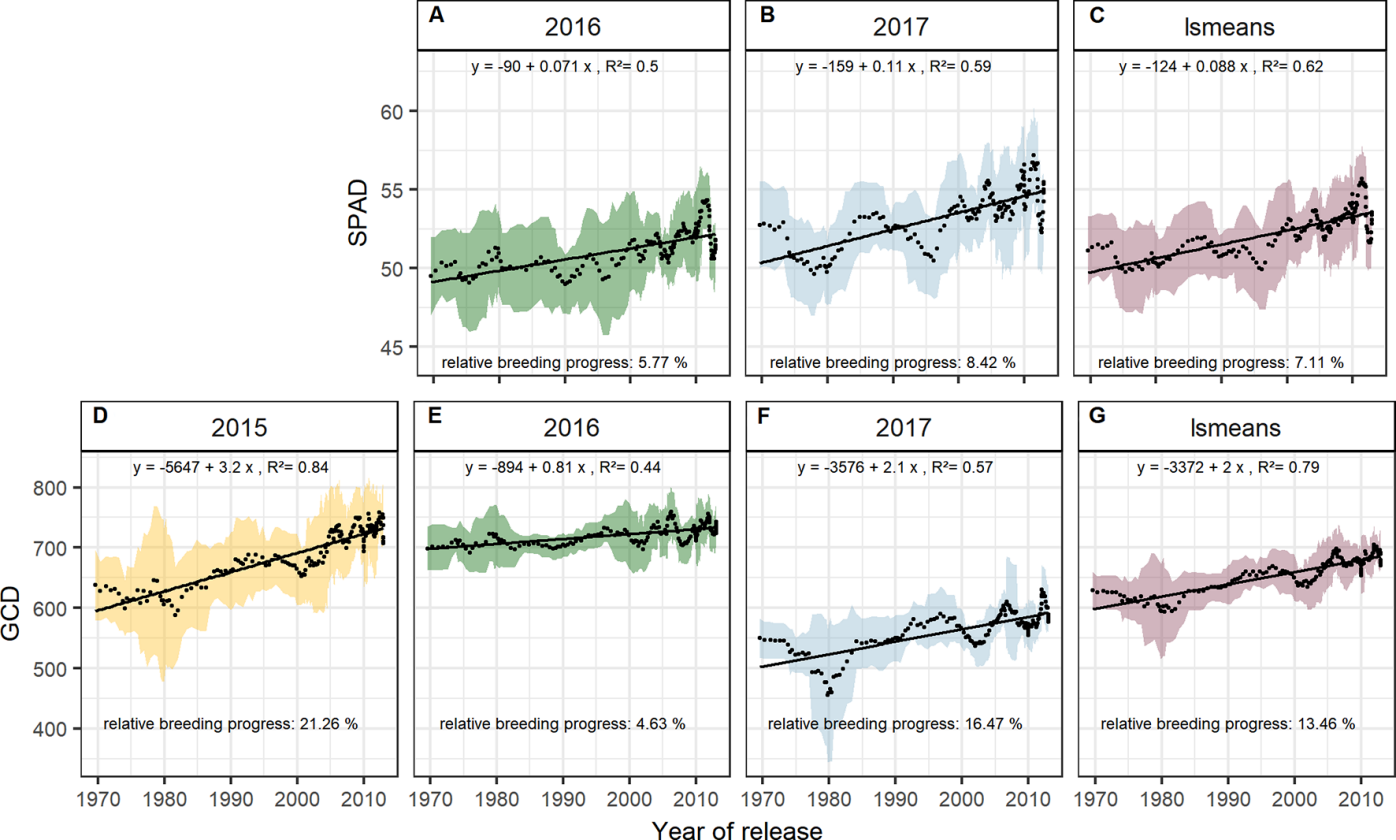
Sliding window plots showing breeding progress of SPAD **(A–C)** and green canopy duration **(D–G)**. For detail, see the caption of **Figure 4**.

The significant Pearson correlations of sink and source parameters and the year of release averaged over all seasons cover a range from r = 0.78 for grain yield to r = 0.17 for TGW. With that, GCD ranked among the highest values (r = 0.52) and is improved by breeding with a relative breeding progress of 13% (**Figure S4A**, **Table 1**). A significant breeding progress was also observed for the photosynthetic activity during anthesis (SPAD) (**Figures 7A–C**).

Breeding progress in LAI_max_ was inconsistent between years (**Figure S4**, **Table 1**), significant only in 2016 (11.4%) and 2017 (−3.7%). Furthermore, in all seasons the absolute breeding progress in LAI_max_ was marginal, indicating that the breeding progress in source capacity was in general not achieved by the increasing canopy size. By contrast, with 17.5% the relative average breeding progress for leaf area duration was the second-highest breeding progress value after grain yield and grain number increase (LAD, **Table 1**). Notwithstanding, the correlation of the year of release and GCD was significant in all seasons and comparable with that for grains per spike (r = 0.52, r = 0.48, respectively, **Figure S4A**). The absolute breeding progress of GCD was about 2 °Cd per year of release (**Table 1**). Thus, modern cultivars stay about 7 days with a mean temperature of 15°C longer green than old cultivars. Although GCD explained the variations in yield to the same extend as grains per spike, breeding progress in GCD showed four times differences between years (**Figures 7D–G**) whereas the breeding progress in grains per spike was independent of the year (about 0.17 grains per year of release, or 6.4 grains per spike from 1970 to 2010, **Table 1**). This implies a higher environmental dependency of canopy duration.

### Significant Marker-Trait Associations for GCD and Biomass

**Figure 8:**
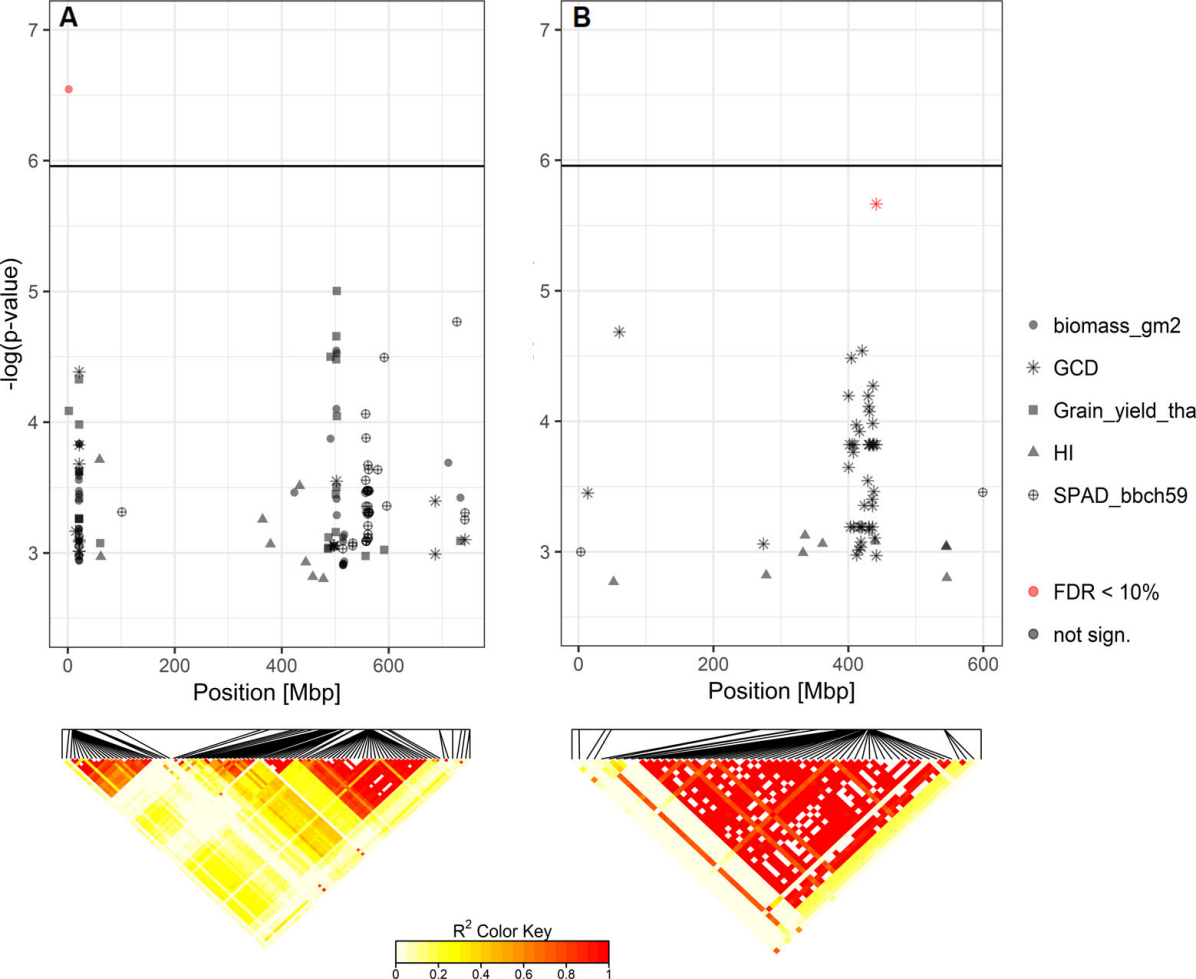
Heat maps of pairwise LD of the SNP-markers with significant marker-trait-associations for **(A)** biomass on chromosome 3A and **(B)** green canopy duration (GCD) on chromosome 6A. SNP-markers among the top 100 -log(p-values) for each analyzed trait on these chromosomes are also shown.

**Figure 9:**
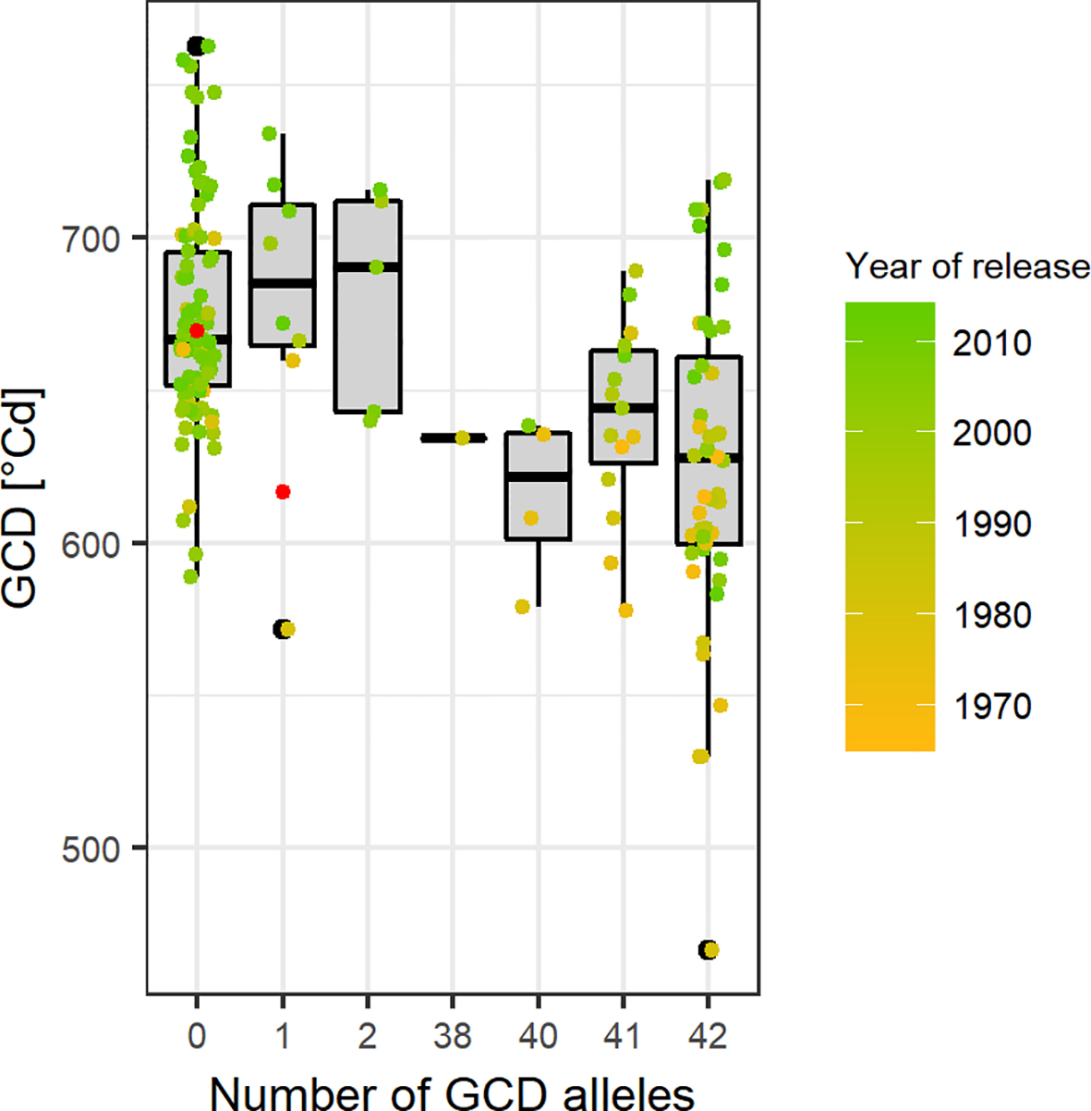
Green canopy duration (GCD) of cultivars in relation to their number of GCD alleles in the selected region between 400 and 441 Mbp on chromosome 6A. Point colors representing the year of release and gray points are the cultivars with unknown year of release. Red points indicate the mean GCD for cultivars carrying the major allele (x = 0) or the minor allele (x = 1).

Based on the Bonferroni threshold with p < 0.05 (-log_10_(p)= 5.96), one significant marker was detected for biomass on chromosome 3A (1.93 Mbp) and considering the FDR < 0.1, one additional significant association appears on chromosome 6A for GCD (441.4 Mbp) (**Figures S6** and **8**). To investigate these signals, the linkage disequilibrium (LD) pattern of all marker-trait associations among the 100 highest -log(p-values) for each analyzed trait were investigated. Chromosomes 3A and 6A showed relevant patterns (**Figure 8**). Besides the significant marker around 2 Mbp for biomass on chromosome 3A, a collection of marker-trait-associations for grain yield and biomass were detected around 500 Mbp with high LD values. Remarkably, several marker-trait associations for grain yield and GCD colocalized at 20 Mbp and showed high LD values. Furthermore, a block of SNP-markers in high LD was observed 560 Mbp which were associated with SPAD at heading stage and at the same time grain yield or biomass (**Figure 8A**). On chromosome 6A we detected a block of SNP markers between 400 and 442 Mbp associated with GCD which were all in high LD (r² = 0.84 for SNP markers between 400 and 442 Mbp). Each of the minor alleles of the GCD associated markers on chromosome 6A had a negative effect on canopy longevity, and the explained phenotypic variance reached up to 22.5% (data not shown). Interestingly, neighboring SNP markers associated with HI or SPAD, were not genetically linked to the GCD markers on this chromosome (**Figure 8B**). However, the effect of the associated SNP markers between 400 and 442 Mbp was not additive, as indicated in **Figure 9**. The mean value for cultivars carrying all 42 minor GCD alleles was not lower than the mean GCD values for cultivars carrying the minor GCD allele with the highest log(p-value). Furthermore, it could be shown that cultivars with no minor GCD allele were rather recently released and the cultivars carrying all minor GCD alleles with negative effects were on average older. This indicates a shift of the frequency of the GCD reducing alleles in that region during breeding history.

## Discussion

### Significant Breeding Progress in Biomass, HI, Grains Per Spike, SPAD, and GCD

The present investigations revealed biomass, HI, grains per spike, SPAD values around anthesis, and GCD as the most relevant traits for progress in German winter wheat breeding in the past five decades. The results are consistent with the findings showing an increased number of grains per spike in modern German cultivars (Würschum et al., 2018). Several other studies also identified grains per spike as the trait with the closest relationship to yield progress in other regional cultivar collections (Brancourt-Hulmel et al., 2003; Acreche et al., 2008; Foulkes et al., 2009; Sanchez-Garcia et al., 2013). This implies that breeding progress has been achieved predominantly by increasing sink size. An important role of sink size was also suggested by studies indicating that HI (Brancourt-Hulmel et al., 2003; Lo Valvo et al., 2018) or TGW (Sadras and Lawson, 2011; Tian et al., 2011; Aisawi et al., 2015) were the main factors of yield increase. However, source characteristics like photosynthetic capacity or canopy longevity, the stay green traits, have also been associated with breeding progress (Fischer et al., 1998; De Vita et al., 2007; Tian et al., 2011; Sanchez-Garcia et al., 2015). The identification of different causal agents for yield progress can on the one hand be assigned to the origin of the cultivars in focus. The geographic pattern in genetic diversity has been demonstrated using 407 cultivars with European origin (Würschum et al., 2018). Among the set of varieties, patterns of allele frequencies matched with the geographical origin of the cultivars. On the other hand, the investigation of the breeding progress is largely dependent on the variance of the cultivars’ year of release in the collection. The studies, elaborating HI as most relevant for breeding progress included cultivars from prior to the Green Revolution (Brancourt-Hulmel et al., 2003; Lo Valvo et al., 2018).

Interestingly, the breeding progress in the present materials accelerated around 1996 especially for grains per spike and the SPAD values (**Figures 6A–D** and **7A–C**). Segmented regressions for grains per spike suggested breakpoints in 2003, 1996, 1996, and 1997 for the seasons 2015, 2016, 2017, and on average, respectively (data not shown). Breeding progress in the first phase was insignificant (or even negative) and steeply increased in the second phase. The slope of the sliding window plots after the breakpoint is more than double (on average: 0.4, r² = 0.68). This indicates that breeding progress in the number of grains per spike started from the middle of 1990s. Nonetheless, the goodness of fit (r² of the regression) improves only slightly with the segmented regression (data not shown). Possible candidates introducing the beneficial genetic materials are two cultivars outnumbering their contemporaries in grains per spike, ‘*Flair*’ and ‘*Dekan*,’ released in 1996 and 1999, respectively. The stepper increase could as well be attributed to a drop of the trait expression before the breakpoint. Two cultivars with conspicuous constant low number of grains per spike were ‘*Asketis*’ and ‘*Aristos*,’ released 1997 and 1998, respectively. Interestingly, these two cultivars have a very similar pedigree and come from the same breeder. The steeper increase or drop in grains per spike, was not translated into the total grain yield. For ‘*Asketis*’ and ‘*Aristos*’ in fact a constantly higher TGW was observed in comparison to other cultivars released in 1997 and 1998, compensating for the reduced number of grains. For SPAD, the parallel development with grains per spike was apparent but the segmented regression did not better explain the breeding progress than the linear regression.

### GCD and Grain Yield: Correlation Does Not Imply Causation

An extended duration of the green leaf area was assumed to boost grain filling and therefore thousand grain weight (Foulkes et al., 2009), but no relationship was observed between the TGW and the source components. So, our hypothesis was rejected. GCD and LAD did not correlate with TGW, but with biomass, HI, grains per m², and total grain yield. An alternative hypothesis could be that, prior to grain filling, at the time point when the number of grains is fixed, the potential longevity of the green canopy is also predetermined. To fill a higher number of grains, the available photosynthetic tissue had to be adjusted either in size, specific activity, or duration. Apparently, an extended canopy life was the most appropriate adjustment in the selection process of German winter wheat breeding. Therefore, the effect of grains per spike on total grain yield was in parts mediated by the extended canopy longevity. Similarly, in the study of Liang et al., 2018, the degree of senescence during grain filling was also negatively correlated to yield but not to thousand grain weight. The authors revealed that the photosynthesis at these stages is determined by the size of the carbon sink which is genetically predefined. The suggestion for wheat breeders was subsequently to select for higher grain number, which is likely to come along with a prolongation of the ability to fill the grains (Liang et al., 2018). But besides leaf longevity, also the photosynthetic capacity is relevant for grain yield as shown here with the results of the SPAD measurements. Growth conditions around anthesis, the stage at which the final number of fertile florets is set, could directly be linked to the number of seeds and therefore grain yield.

### Relevance of GCD for Breeding

Grain number per spike is in detail determined by the number of spikelets per spike and the number of grains per spikelet, namely spikelet fertility. In-depth analyses of the spike and grain traits identified the spikelet fertility as the key driver of grain yield progress in wheat (Würschum et al., 2018). It was further suggested that the trait was unintentionally selected during breeding progress of German winter wheat and therefore holds a high potential if breeders start to actively select (Würschum et al., 2018). Our study presents GCD as a hidden mediator of yield potential. It seems that canopy longevity was unintentionally increased by selection processes during the last 50 years of breeding history and therefore holds potential for further progress by targeted selection. Furthermore, GCD can be assessed easily and is suitable for large scale phenotyping.

To develop higher yielding varieties, selecting parental lines based on physiological characteristics for complementary crosses is one promising strategy. Additionally, it has been suggested to set a focus during the selection cycles on the yield components, because of higher heritabilities in comparison to yield itself (Falconer and Mackay, 2009; Schulthess et al., 2017). Plant breeders might use the information about the drivers of the historical breeding progress as one criterion in their strategy to obtain further improved wheat varieties.

The persistence of the green canopy together with the photosynthetic capacity have been suggested as target traits in the process of improving the radiation use efficiency (RUE) by applying high-throughput phenotyping techniques. Aerial imaging is proposed as a promising strategy to estimate the canopy photosynthesis and thereby light utilization on a spatial and temporal scale by multispectral-sensing. The advancement of these techniques will, together with genomics, facilitate a more efficient selection of source parameters and thereby accelerate progress in the final yield (Furbank et al., 2019).

### Genetic Associations of GCD

Previous studies on the genetics of stay-green traits in winter wheat were exclusively designed to investigate genetic variation with experiments under different drought and heat stress conditions. A prolongation of the available photosynthetic tissue is known to facilitate yield formation under post-anthesis abiotic stress conditions without detrimental impact under non-stress conditions (Verma et al., 2004; Kumar et al., 2010; Vijayalakshmi et al., 2010; Christopher et al., 2014; Pinto et al., 2016; Shi et al., 2017; Christopher et al., 2018). The present study, however, shows that GCD even has positive effects on yield under rainfed conditions and optimal crop management.

QTLs of GCD explained up to 22% of the phenotypic variance and at only one single location the SNP markers exceeded the significance threshold (chromosome 6A). Nevertheless, this dataset showed the progress of GCD during breeding history genetically, in parallel to previous findings demonstrating a shift in haplotype blocks with detrimental effects on stay-green through breeding (Voss-Fels et al., 2019a). The confirmation of the trait association with this genomic region proves the potential of the novel haplotype-based approach, where complete chromosomal segments instead of single markers are applied for the association analyses. The expected colocalization of significant association with source and sink traits could not confirmed in the present study. SNP markers, significantly associated to sink or source traits, were not linked, indicated by a low LD. To confirm the co-evolution genetically, the LD was expected to increase within the genomic regions of interest with the year of release. However, the average LD decrease instead. For the region on chromosome 6A (400–442 Mbp), the LD decreased from r² = 0.79 within a cultivar group released before 1970 to r² = 0.68 within a cultivar group released after 2010. The selection pressure against early senescing phenotypes possibly has favored recombination in this particular region. Interestingly, within the other conspicuous regions on chromosome 3A, the LD did not change and also the complete LD per chromosome calculated based on all SNP markers on each chromosome did rather decrease with the year of release for nearly all chromosomes (except 1D, 3D, and 4D) and was on average low (0.10 for cultivars release before 1970 and 0.07 for cultivars released after 2010).

To our knowledge, there are only two chromosomes, on which no one ever detected a significant marker trait association with a stay-green trait in wheat: 5D and 6D. All other 19 chromosomes have been mentioned to carry some genetic regions relevant for the stay-green trait expression but unfortunately, there is no chromosome, which was conspicuous in all genomic marker association reports. This indicates the great complexity of the trait. Regions on chromosomes 1B and 3B are most prominent as in summary four further groups of researchers detected relevant signals (Kumar et al., 2010; Vijayalakshmi et al., 2010; Naruoka et al., 2012; Pinto et al., 2016; Shi et al., 2017; Christopher et al., 2018). Contrary to the present work, the previous studies used solely mapping populations, consisting of double haploids (Verma et al., 2004; Shi et al., 2017; Christopher et al., 2018) or recombinant inbred lines (Kumar et al., 2010; Vijayalakshmi et al., 2010; Naruoka et al., 2012; Pinto et al., 2016) which were all obtained from crosses of cultivars contrasting in the stay-green and senescence traits. The stay-green phenotypes were examined in many cases via measurements of the normalized difference vegetation index but also visual scorings or measurements of the chlorophyll content with the SPAD-502 meter are common. Most of the investigators fitted sigmoid curves to the data but with slightly different formulae so that and the estimated parameters were different. Either the integral, time points or durations were used for marker trait associations. Nevertheless, the studies hold potential to be of great use for marker assisted breeding, when the genetic map positions get synchronized on the physical reference map, as most previous studies published the genetic QTL position. Additionally, further work is needed to investigate possible underlying candidate genes and the allele frequency changes to further resolve patters of selection and linkage. Nevertheless, the necessity to understand the genetic bases of source related traits was again emphasized in recent investigations on the sink strength, as the increase in spikelets per spike can only be translated into considerable higher yield, when the source is adapted concomitantly (Kuzay et al., 2019).

## Conclusion

The present study identified the photosynthetic activity around anthesis and the longevity of the green canopy as the relevant source traits ensuring the supply to the increased number of sink organs in the course of wheat breeding. The linkage between duration and capacity of the source and grain number suggests a predetermined longevity of green leaf area around anthesis when the grain number is fixed. Our results suggest placing emphasis on a balanced improvement of floret fertility and canopy longevity during the development of wheat cultivars. Furthermore, combining and selecting the most promising components of sink and source traits may further increase grain yield. The genome wide association study underpinned the association of breeding progress in canopy longevity. Further analyses of allele frequencies and associations with known genes involved in plant development will reveal in depth insights in the interdependencies of the yield relevant traits and whether the theory of unintentional selection can be confirmed.

## Data Availability Statement

The BLUE-values of the genotypes used in this manuscript are available in ZENODO data repository (Voss-Fels et al., 2019b). The 135K SNP-data are available in the **Supplementary Information**.

## Author Contributions

The study was conceived by CL and HS. The phenotypic data was generated by CL and T-WC. CL performed the analyses, drafted the manuscript and discussed with T-WC, AS, and HS. All authors have read and approved the final manuscript.

## Funding

This research was funded by the German Federal Ministry of Education and Research (BMBF) grant 031A354 provided to HS within the project Breeding Innovations in Wheat for Resilient Cropping Systems (BRIWECS) as part of the funding initiative Innovative Plant Breeding in the Production Systems (IPAS). We thank Leibniz Open Access Publishing Fund for covering the publication cost.

## Conflict of Interest

The authors declare that the research was conducted in the absence of any commercial or financial relationships that could be construed as a potential conflict of interest.
