# Supplementary material for: Co-Evolution of Sink and Source in the Recent Breeding History of Winter Wheat in Germany
Source: Front Plant Sci. 2020 Feb 7;10:1771. doi: 10.3389/fpls.2019.01771 (PMC7019858; doi:10.3389/fpls.2019.01771)
Supplement: Supplementary file 1 [file DataSheet_1.docx]

# **Supplementary figure**


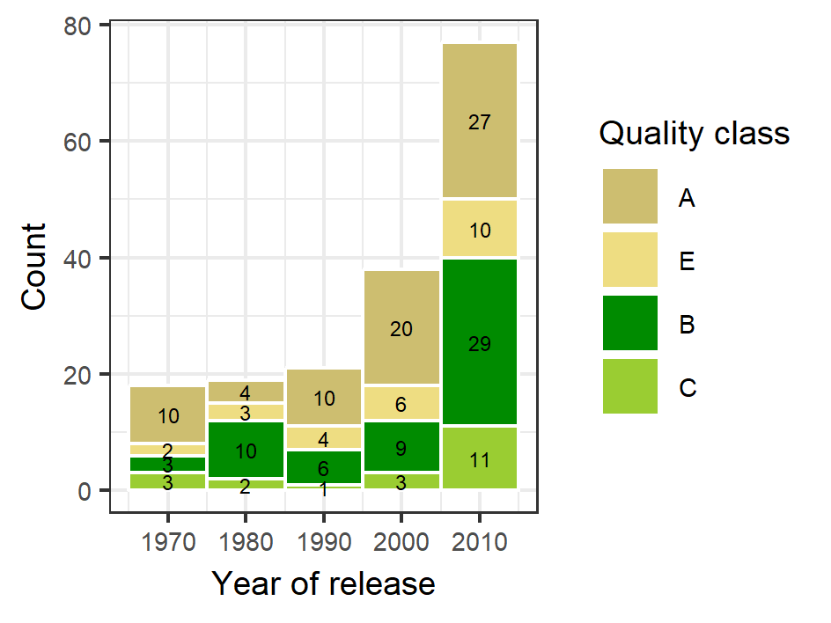


## Fig. S1: Proportion of cultivars per decade and quality class within the cultivar collection representing the breeding history of German winter wheat, oldest cultivar from 1966 and youngest from 2013, in total 174 cultivars including 5 hybrids.

##
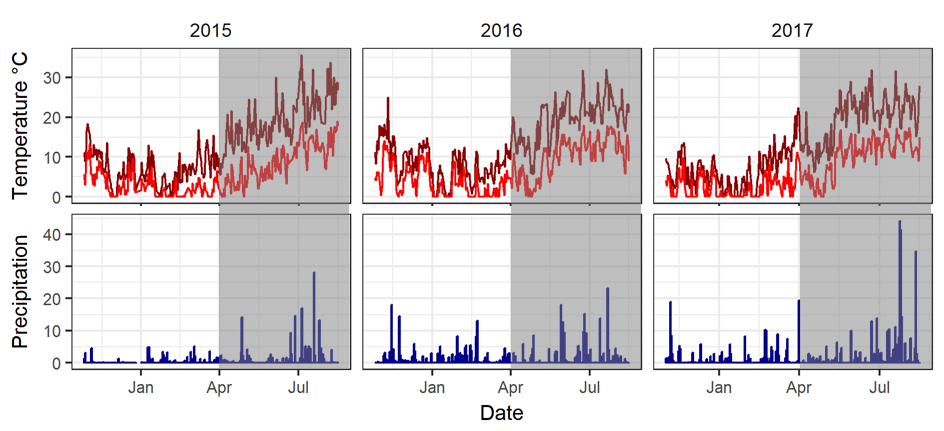


Fig. S2: Summary of minimum (above 0°C) and maximum temperature and daily precipitation sum during the experimental periods, grey area: approximately generative phase.


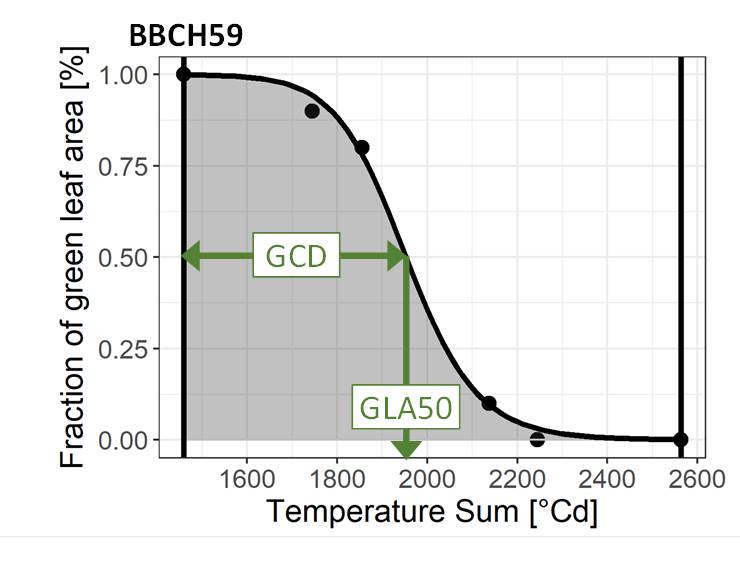
Fig. S3: Explanation graph for parameters describing the senescence pattern. The black dots are visually scored green leaf area (%). A logistic power function (black line) with two parameters was fitted to the data. For each cultivar, integral of green canopy area (grey area) was calculated between its heading (BBCH59) and harvest date in each season. GLA50 is the temperature sum when 50% green was reached and green canopy duration (GCD) is the temperature sum from heading to GLA50.


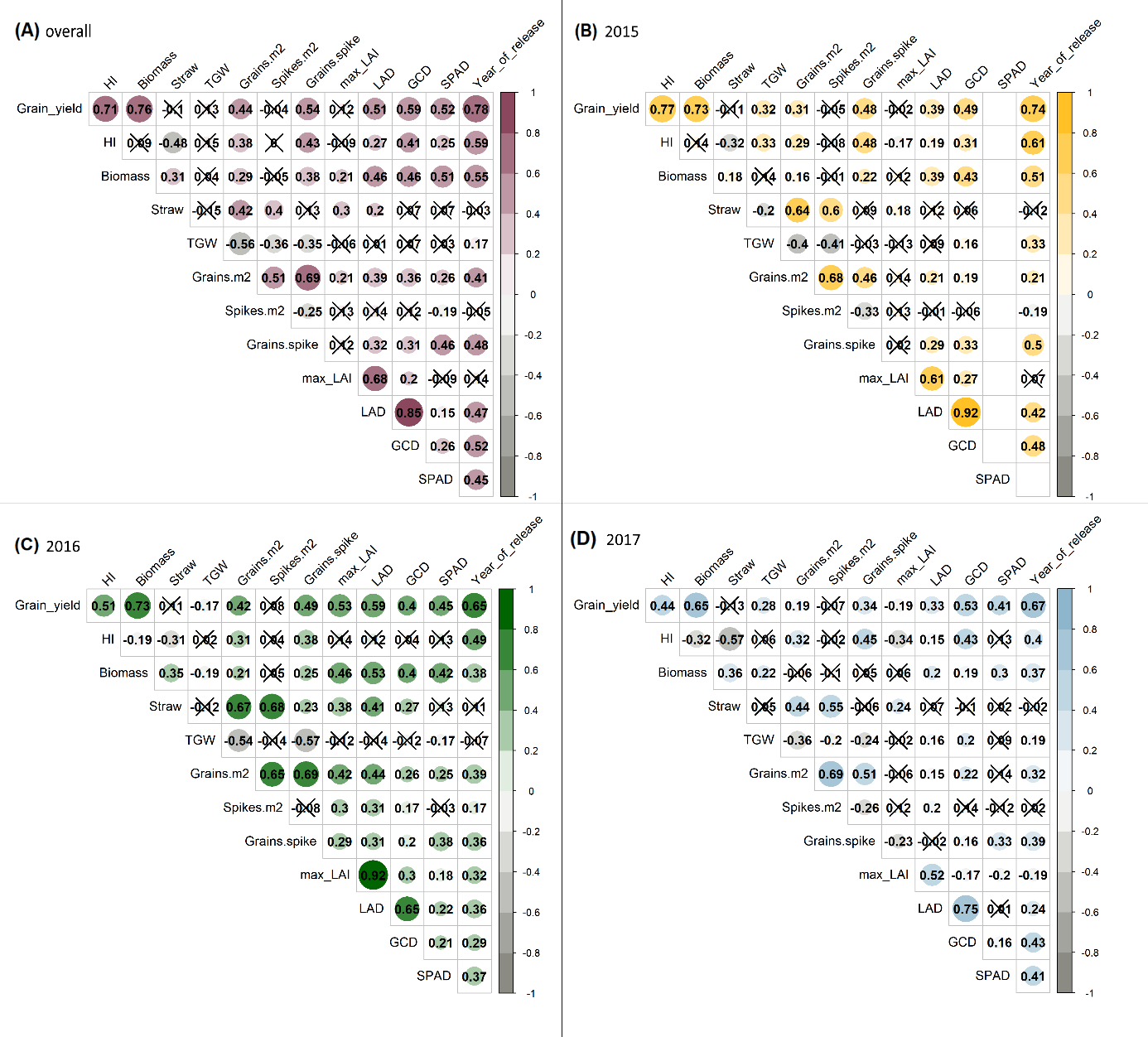


Fig. S4: Pearson correlations of yield and stay green parameters, A: means for cultivars over **all three seasons and two replications each**, B: means for cultivars within **2015**, C: means for cultivars within **2016**, D: means for cultivars within **2017**, crossed values were not significant.


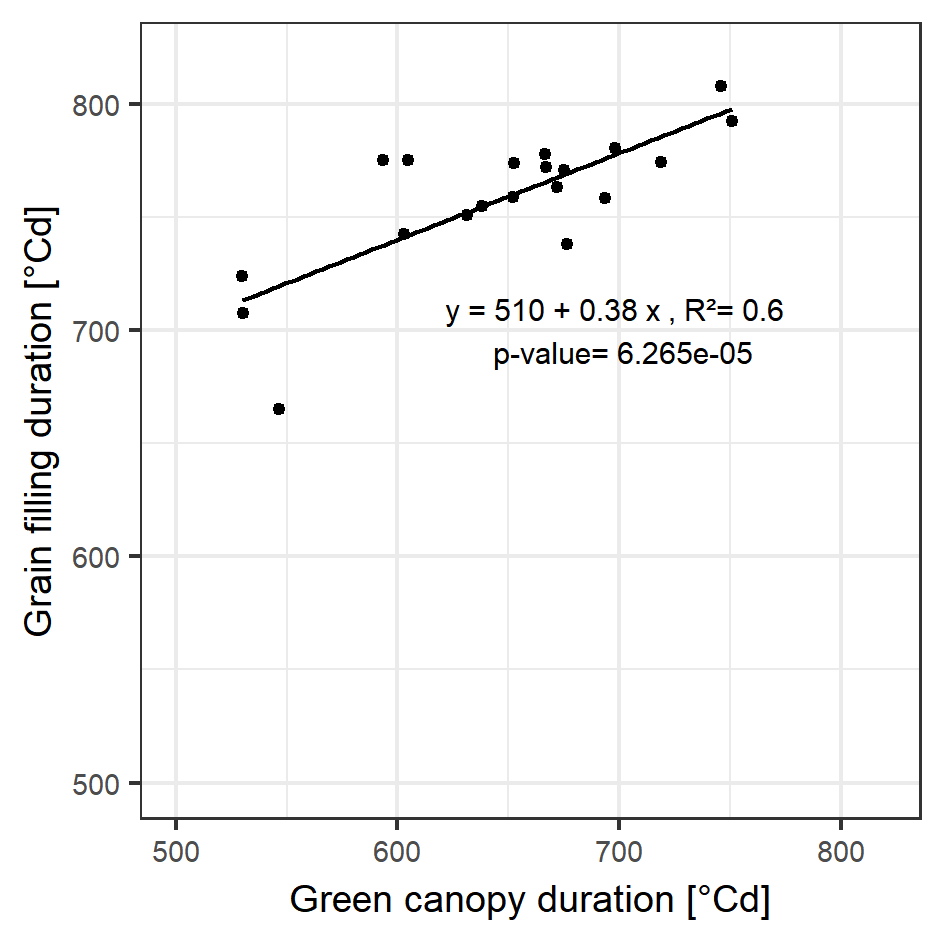


Fig. S5: Linear relationship between the grain filling duration (temperature sum of BBCH87 – temperature sum of BBCH59) and the green canopy duration for a subset of 20 cultivars. Each dot represents a mean value of three seasons and 2 replications (n=6).


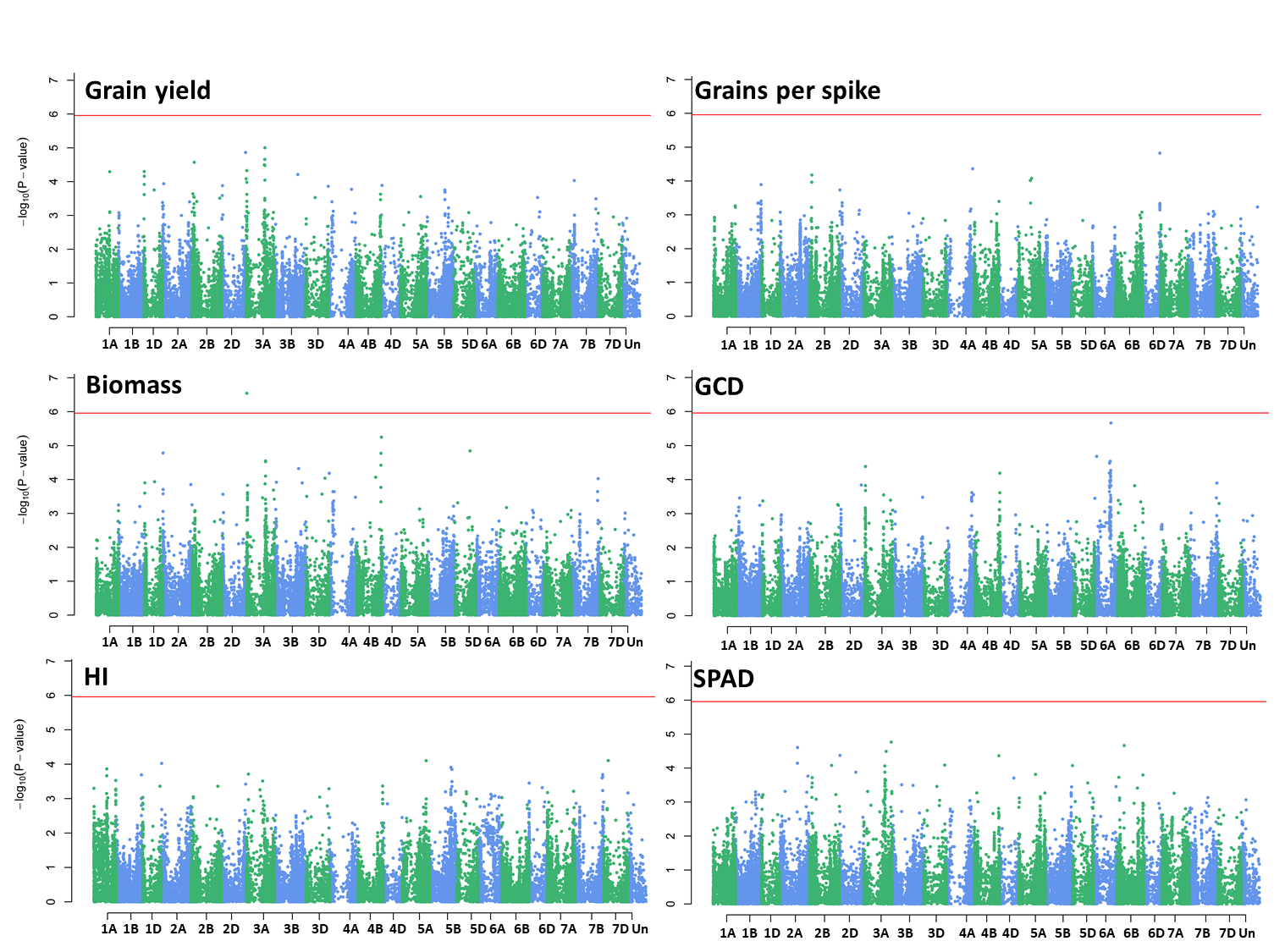


Fig. S6: Manhattan plots for genome wide marker - trait associations. P-values shown on a -log10(p-values) scale and the red line indicates the significance threshold (Bonferroni, 0.05)

Table S1: Complete list of cultivars, Bh= breeding history collection, Hyb= hybrid cultivar, org= cultivars with recommendation for use in organic farming, *= excluded from the breeding history analyses because of remarkable below-average yields for the corresponding year of registration, double bar indicates the distinction between cultivars used for breeding history analyses and the supplementary diverse cultivars for the GWAS

| Cultivar name | Year of reg. | Breeder or representative in Germany according to the respective volume of the German Variety List (BSL) | Qualilty class | Country of registration | sub-group (field) | sub-set (analyses) |
| --- | --- | --- | --- | --- | --- | --- |
| **Diplomat** | **1966** | Firlbeck | A | DE | late, tall | Bh |
| **Pantus** | **1966** | Streng | A | DE | late, short | Bh |
| **Admiral** | **1968** | Firlbeck | A | DE | late, tall | Bh |
| **Caribo** | **1968** | Heidenreich und Eger | B | DE | late, tall | Bh |
| **Progress** | **1969** | Hege, H.-U. | A | DE | late, short | Bh |
| **Kranich** | **1969** | Lochow-Petkus | A | DE | late, short | Bh |
| **Joss** | **1972** | Breustedt | C | DE | late, short | Bh |
| **Topfit** | **1972** | Strube, Dr. H. | B | DE | late, tall | Bh |
| **Benno** | **1973** | Bauer, G. | E | DE | late, tall | Bh |
| **Kormoran** | **1973** | Lochow-Petkus | A | DE | late, tall | Bh |
| **Saturn** | **1973** | MPI | C | DE | late, short | Bh |
| **Disponent** | **1975** | Bayrische Saatzuchtgesellschaft | A | DE | late, tall | Bh |
| **Monopol** | **1975** | Firlbeck | E | DE | late, tall | Bh |
| **Nimbus** | **1975** | Firlbeck | B | DE | early, short | Bh |
| **Vuka** | **1975** | Franck | A | DE | late, tall | Bh |
| **Carimulti** | **1975** | Heidenreich und Eger | C | DE | late, tall | Bh |
| **Carisuper** | **1975** | Heidenreich und Eger | A | DE | late, tall | Bh |
| **Maris Huntsman** | **1975** | Nordsaat Saatzucht | A | DE | late, short | Bh |
| **Götz** | **1978** | Bayrische Saatzuchtgesellschaft | B | DE | late, short | Bh |
| **Kobold** | **1978** | Firlbeck | B | DE | late, tall | Bh |
| **Aquila** | **1979** | Nickerson | C | UK, IT | late, tall | Bh |
| **Tabor** | **1979** | Strube, Dr. H. | A | DE | late, short | Bh |
| **Camp Remy** | **1980** | Unisigma | B | DE | early, short | Bh |
| **Severin** | **1980** | Bauer, G. | E | DE | late, short | Bh |
| **Urban** | **1980** | Bauer, G. | E | DE | late, short | Bh |
| **Kronjuwel** | **1980** | Bayrische Saatzuchtgesellschaft | B | DE | late, short | Bh |
| **Rektor** | **1980** | Firlbeck | E | DE | late, tall | Bh |
| **Basalt** | **1980** | Hege, H.-U. | B | DE | late, tall | Bh |
| **Kanzler** | **1980** | Saatzucht Engelen Büchling e.K. | B | DE | late, tall | Bh |
| **Oberst** | **1980** | Saatzucht Engelen Büchling e.K. | A | DE | late, tall | Bh |
| **Granada** | **1980** | Saatzucht Schweiger GbR | B | DE | late, short | Bh |
| **Sperber** | **1982** | Lochow-Petkus | A | DE | late, short | Bh |
| **Kraka** | **1982** | Petersen, A.S. | A | DE | late, tall | Bh |
| **Ares** | **1983** | Strube, Dr. H. | B | DE | late, tall | Bh |
| **Apollo** | **1984** | Saatzucht Josef Breun | C | DE | late, tall | Bh |
| **Knirps** | **1985** | Semundo | B | DE | late, tall | Bh |
| **Sorbas** | **1985** | Strube, Dr. H. | B | DE | late, short | Bh |
| **Herzog** | **1986** | Saatzucht Josef Breun | A | DE | late, short | Bh |
| **Alidos** | **1987** | Saatzucht Hadmersleben | E | DE | late, tall | Bh |
| **Obelisk** | **1987** | Strube, Dr. H. | B | NL, DE | late, short | Bh |
| **Orestis** | **1988** | Strube, Dr. H. | B | DE | late, short | Bh |
| **Greif** | **1989** | Lochow-Petkus | B | DE | late, short | Bh |
| **Zentos** | **1989** | Saatzucht Hadmersleben | E | DE | late, tall | Bh |
| **Astron** | **1989** | Strube, Dr. H. | A | DE | late, tall | Bh |
| **Kontrast** | **1990** | Saatzucht Hadmersleben | A | DE | early, short | Bh |
| **Contra** | **1990** | Saatzucht Josef Breun | C | DE | late, short | Bh |
| **Toronto** | **1990** | Strengs Erben | A | DE | late, short | Bh |
| **Konsul** | **1990** | SW Seeds | B | DE | late, tall | Bh |
| **Ibis** | **1991** | Lochow-Petkus | A | DE | late, tall | Bh |
| **Tarso** | **1992** | Saatzucht Hadmersleben | A | DE | late, short | Bh |
| **Aron** | **1992** | Semundo | E | DE | late, tall | Bh |
| **Ritmo** | **1993** | Cebeco | B | DE | late, short | Bh |
| **Tambor** | **1993** | Semundo | A | DE | late, tall | Bh |
| **Piko** | **1994** | Nordsaat Saatzucht | B | DE | early, short | Bh |
| **Transit** | **1994** | Saatzucht Josef Breun | A | DE | late, short | Bh |
| **Pegassos** | **1994** | Strube, Dr. H. | A | AT, CH, DE, LT, PL, SI, SK | early, short | Bh |
| **Isengrain** | **1996** | Florimond-Desprez | B | FR, SI, ES | early, short | Bh |
| **Flair** | **1996** | Saatzucht Schweiger GbR | B | DE | late, short | Bh |
| **Apache** | **1997** | Nickerson | A | CZ | early, short | Bh |
| **Aristos** | **1997** | Strube, Dr. H. | A | DE | late, tall | Bh |
| **Ludwig** | **1998** | Probstdorfer Saatzucht | A | AT, DE, CZ, HR, PL, HU and SI | late, tall | Bh |
| **Cardos** | **1998** | Saatzucht Hadmersleben | A | DE | early, short | Bh |
| **Asketis** | **1998** | Strube, Dr. H. | A | DE | late, tall | Bh |
| **Dekan** | **1999** | Lochow-Petkus | B | DE | late, short | Bh |
| **Drifter** | **1999** | Nickerson | B | DE | late, short | Bh |
| **Skater** | **2000** | Limagrain GmbH | B | DE | late, short | Bh |
| **Biscay** | **2000** | Lochow-Petkus | C | DE | late, short | Bh |
| **Magnus** | **2000** | Saatzucht Engelen Büchling e.K. | A | DE | late, tall | Bh |
| **Altos** | **2000** | Syngenta Seeds GmbH | E | DE | late, short | Bh |
| **Terrier** | **2001** | Nickerson | B | DE | late, short | Bh |
| **Sokrates** | **2001** | Saatzucht Engelen Büchling e.K. | A | DE | late, tall | Bh |
| **Winnetou** | **2002** | Firlbeck | C | DE | late, short | Bh |
| **Cubus** | **2002** | Lochow-Petkus | A | DE | late, short | Bh |
| **Tommi** | **2002** | Nordsaat Saatzucht | A | DE | late, short | Bh |
| **Ellvis** | **2002** | Saatzucht Josef Breun | A | DE | late, short | Bh |
| **Enorm** | **2002** | Saatzucht Schweiger GbR | E | DE | late, short | Bh |
| **SW Topper** | **2002** | SW Seeds | E | DE | late, short | Bh |
| **Akteur** | **2003** | DSV | E | DE | late, tall | Bh |
| **Limes** | **2003** | Innoseeds | B | DE | late, short | Bh |
| **Paroli** | **2004** | DSV | A | DE | late, short | Bh |
| **Türkis** | **2004** | SW Seeds | A | DE | late, short | Bh |
| **Magister** | **2005** | Bauer, G. | E | DE | late, short | Bh |
| **Chevalier** | **2005** | DSV | A | AT, CZ, LT, LU | late, short | Bh |
| **Anthus** | **2005** | KWS Lochow GmbH | B | DE | late, short | Bh |
| **Tuareg** | **2005** | Nordsaat Saatzucht | A | DE | late, short | Bh |
| **Schamane** | **2005** | Saatzucht Engelen Büchling e.K. | A | DE | late, short | Bh |
| **Impression** | **2005** | Saatzucht Schweiger GbR | A | DE | late, short | Bh |
| **Brillant** | **2005** | SW Seeds | A | DE | late, short | Bh |
| **Torrild** | **2005** | W. von Borries-Eckendorf | A | DE | late, short | Bh |
| **Carenius** | **2006** | Dieckmann | B | DE | late, short | Bh |
| **Potenzial** | **2006** | DSV | A | DE | late, short | Bh |
| **Skalmeje** | **2006** | KWS Lochow GmbH | C | DE | late, short | Bh |
| **Mulan** | **2006** | Nordsaat Saatzucht | B | DE | late, short | Bh |
| **Premio** | **2006** | RAGT | B | FR | early, short | Bh |
| **Manager** | **2006** | Saatzucht Schweiger GbR | B | DE | late, short | Bh |
| **Lucius** | **2006** | Secobra Recherches | A | DE | late, short | Bh |
| **Zobel** | **2006** | Syngenta Seeds GmbH | A | DE | late, short | Bh |
| **Skagen** | **2006** | W. von Borries-Eckendorf | E | DE | late, short | Bh |
| **Jenga** | **2007** | Ackermann Saatzucht | A | DE | late, short | Bh |
| **Boregar** | **2007** | RAGT | A | FR | early, short | Bh |
| **Esket** | **2007** | RAGT | A | DE | late, short | Bh |
| **Inspiration** | **2007** | Saatzucht Josef Breun | B | DE | late, short | Bh |
| **Fedor** | **2007** | W. von Borries-Eckendorf | A | DE | late, short | Bh |
| **Profilus** | **2008** | RAGT | A | DE | late, short | Bh |
| **JB Asano** | **2008** | Saatzucht Josef Breun | A | DE | late, short | Bh |
| **Jafet** | **2008** | Sandra Senghaas-Kirschenlohr | E | DE | late, short | Bh |
| **Tabasco** | **2008** | W. von Borries-Eckendorf | C | DE | late, short | Bh |
| **Zappa** | **2009** | Ackermann Saatzucht | C | DE | late, short | Bh |
| **Primus** | **2009** | DSV | B | DE | late, short | Bh |
| **Kredo** | **2009** | Nordsaat Saatzucht | B | DE | late, short | Bh |
| **Global** | **2009** | RAGT | B | DE, AT | late, short | Bh |
| **Event** | **2009** | Saatzucht Josef Breun | E | DE | late, short | Bh |
| **Arktis** | **2010** | DSV | E | DE | late, short | Bh |
| **Matrix** | **2010** | DSV | B | DE | late, short | Bh |
| **Muskat** | **2010** | DSV | C | DE | early, short | Bh |
| **KWS Pius** | **2010** | KWS Lochow GmbH | A | DE | late, short | Bh |
| **Edgar** | **2010** | Limagrain GmbH | B | DE | late, short | Bh |
| **Kalahari** | **2010** | Limagrain GmbH | B | DE, BE | late, short | Bh |
| **Linus** | **2010** | RAGT | A | DE | late, short | Bh |
| **Oxal** | **2010** | RAGT | B | DE | late, short | Bh |
| **Orcas** | **2010** | Secobra Recherches | B | DE | early, short | Bh |
| **Alves** | **2010** | SW Seeds | A | DE | late, tall | Bh |
| **KWS Santiago** | **2011** | KWS Lochow GmbH | C | UK | late, short | Bh |
| **Colonia** | **2011** | Limagrain GmbH | B | DE, BE, HU | late, short | Bh |
| **Intro** | **2011** | RAGT | B | DE, FR DE, FR | late, short | Bh |
| **Kometus** | **2011** | Saatzucht Schweiger GbR | A | DE | late, short | Bh |
| **Nelson** | **2011** | Saatzucht Schweiger GbR | E | DE | late, short | Bh |
| **Xanthippe** | **2011** | Sejet Planteforaedling I/S | C | DE | late, short | Bh |
| **Glaucus** | **2011** | Strube, Dr. H. | A | DE | late, short | Bh |
| **Tobak** | **2011** | W. von Borries-Eckendorf | B | DE | late, short | Bh |
| **Joker** | **2012** | DSV | A | DE | late, short | Bh |
| **Patras** | **2012** | DSV | A | DE | late, short | Bh |
| **KWS Ferrum** | **2012** | KWS Lochow GmbH | B | DE | early, short | Bh |
| **Atomic** | **2012** | Limagrain GmbH | A | DE | late, short | Bh |
| **Capone** | **2012** | Limagrain GmbH | A | DE | late, short | Bh |
| **Forum** | **2012** | Nordsaat Saatzucht | A | DE, EE, PL, SE | late, short | Bh |
| **Mentor** | **2012** | RAGT | B | DE | late, short | Bh |
| **WW 4180** | **2012** | Saatzucht Josef Breun | NA | DE | early, short | Bh |
| **Bombus** | **2012** | Secobra Recherches | C | DE | late, short | Bh |
| **Estivus** | **2012** | Strube, Dr. H. | A | DE | late, short | Bh |
| **SY Ferry** | **2012** | Syngenta Seeds GmbH | B | DE | late, short | Bh |
| **Boxer** | **2013** | Ackermann Saatzucht | C | DE | late, tall | Bh |
| **KWS Cobalt** | **2013** | KWS Lochow GmbH | A | DE | late, short | Bh |
| **Kurt** | **2013** | Limagrain GmbH | B | DE | late, short | Bh |
| **Anapolis** | **2013** | Nordsaat Saatzucht | C | DE | late, short | Bh |
| **Rebell** | **2013** | RAGT | A | DE | late, short | Bh |
| **Avenir** | **2013** | Saatzucht Josef Breun | A | DE | late, short | Bh |
| **Gourmet** | **2013** | Secobra Recherches | E | DE | late, short | Bh |
| **Landsknecht** | **2013** | Secobra Recherches | C | DE | late, short | Bh |
| **Memory** | **2013** | Secobra Recherches | B | DE | late, short | Bh |
| **Apertus** | **2013** | Strube, Dr. H. | A | DE | late, short | Bh |
| **Rumor** | **2013** | Strube, Dr. H. | B | DE | early, short | Bh |
| **Desamo** | **2013** | Syngenta Seeds GmbH | B | DE | late, short | Bh |
| **Gordian** | **2013** | Syngenta Seeds GmbH | B | DE | late, short | Bh |
| **Edward** | **2013** | W. von Borries-Eckendorf | B | DE | late, short | Bh |
| **Hybred** | **2003** | Nordsaat Saatzucht | B | DE, FR | early, short | Bh^Hyb^ |
| **Hystar** | **2007** | Saaten Union Recherche | B | FR | early, short | Bh^Hyb^ |
| **Hyland** | **2009** | Nordsaat Saatzucht | B | DE, HU | early, short | Bh^Hyb^ |
| **Hybery** | **2010** | Saaten Union Recherche | B | FR | early, short | Bh^Hyb^ |
| **Hylux** | **2012** | Saaten Union | B | FR | early, short | Bh^Hyb^ |
| **Bussard** | **1990** | Lochow-Petkus | E | DE | late, tall | Bh^Org^ |
| **Batis** | **1994** | Strube, Dr. H. | A | DE | late, tall | Bh^Org^ |
| **Tiger** | **2001** | Franck | A | DE | late, tall | Bh^Org^ |
| **Hermann** | **2004** | Nickerson | C | DE | late, short | Bh^Org^ |
| **Kerubino** | **2004** | Saatzucht Schmid Landau | E | DE | late, short | Bh^Org^ |
| **Akratos** | **2004** | Strube, Dr. H. | A | DE | late, tall | Bh^Org^ |
| **Discus** | **2007** | Pflanzenzucht SaKa GmbH&Co.KG | A | DE | late, short | Bh^Org^ |
| **Famulus** | **2010** | DSV | E | DE | late, short | Bh^Org^ |
| **Florian** | **2010** | Nordsaat Saatzucht | E | DE | late, short | Bh^Org^ |
| **Genius** | **2010** | Nordsaat Saatzucht | E | DE | late, short | Bh^Org^ |
| **Meister** | **2010** | RAGT | A | DE | late, short | Bh^Org^ |
| **Elixer** | **2012** | W. von Borries-Eckendorf | C | DE | late, short | Bh^Org^ |
| **Pionier** | **2013** | DSV | A | DE | late, short | Bh^Org^ |
| **KWS Milaneco** | **2013** | KWS Lochow GmbH | E | DE | late, tall | Bh^Org^ |
| Naturastar | 2002 | Saatzucht Schweiger GbR | A | DE | late, tall | Bh^Org*^ |
| Aszita | 2005 | Getreidezüchtung Peter Kunz | B | DE | late, tall | Bh^Org*^ |
| Butaro | 2009 | Landbauschule Dottenfelderhof e.V. | E | DE | late, tall | Bh^Org*^ |
| Soissons | 1987 | Florimond-Desprez | NA | BE, ES, FR, IE, IT, SI | early, short | Div^europ. reg.^ |
| Tremie | 1991 | Serasem | NA | ES, FR, IT | early, short | Div^europ. reg.^ |
| Gaucho | 1993 | USDA-ARS, Oklahoma AES | NA | US | late, short | Div^europ. reg.^ |
| Sponsor | 1994 | Unisigma | NA | FR, IE | late, short | Div^europ. reg.^ |
| Ivanka | 1998 | Inst. of Field and Veg. Crops, Novi Sad | NA | CS | early, short | Div^europ. reg.^ |
| Claire | 1999 | Nickerson | C | IE, UK | late, short | Div^europ. reg.^ |
| Caphorn | 2000 | RAGT | NA | FR | early, short | Div^europ. reg.^ |
| Solstice | 2001 | Limagrain GmbH | NA | UK | late, short | Div^europ. reg.^ |
| Cordiale | 2003 | KWS UK | NA | UK | early, short | Div^europ. reg.^ |
| Robigus | 2004 | KWS UK | B | UK | late, short | Div^europ. reg.^ |
| Einstein | 2004 | Nickerson | B | UK | late, short | Div^europ. reg.^ |
| Alixan | 2005 | Limagrain GmbH | A | FR | early, short | Div^europ. reg.^ |
| Arlequin | 2007 | Limagrain GmbH | NA | FR | early, short | Div^europ. reg.^ |
| Oakley | 2008 | KWS UK | C | UK, BE | late, short | Div^europ. reg.^ |
| Phoenix | 1981 | WWAI | NA | AU; US | early, tall | Div |
| Helios | NA | Arizona Plant Breeders | NA | US | late, short | Div |
| Mex. 3 | NA | BAZ | NA | MX | early, short | Div |
| Cajeme 71 | 1971 | CIMMYT | NA | MX | early, short | Div |
| Mex. 17 bb | NA | CIMMYT | NA | MX | early, short | Div |
| BCD 1302/83 | NA | Goertzen Seed Research | NA | MD | early, short | Div |
| Sonalika | 1971 | Indian Agricultural Research Institute | NA | IN | early, short | Div |
| Lambriego INIA | 1981 | INIA | NA | CL | early, short | Div |
| Siete Cerros 66 | 1966 | CIMMYT | NA | MX | early, short | Div |
| Triple dirk \S\"" | NA | INIA | NA | AU | early, short | Div |
| Pobeda | 1990 | Inst. of Field and Veg. Crops, Novi Sad | NA | SR | early, short | Div |
| Renesansa | NA | Inst. of Field and Veg. Crops, Novi Sad | NA | SR | early, short | Div |
| Centurk | 1971 | Nebraska Agr. Exp. Station | NA | US | early, tall | Div |
| Avalon | 1980 | Plant Breeding Int. Cambridge | NA | GB | early, short | Div |
| Highbury | 1968 | Plant Breeding Int. Cambridge | A | GB | early, short | Div |
| Brigand | 1979 | Plant Breeding Int. Cambridge | NA | GB | late, short | Div |
| TJB 990-15 | NA | Plant Breeding Int. Cambridge | NA | GB | late, short | Div |
| Benni mult. | NA | Purdue University | NA | US | early, tall | Div |
| Hope | 1927 | S.Dakota Agr. Exp. Station | NA | US | early, tall | Div |
| SUR99820 | NA | Saaten Union Recherche | NA | FR | early, short | Div |
| Cappelle Desprez | NA | SAS Florimond Desprez | C | FR; CH; GB | late, tall | Div |
| Durin | 1986 | Plant Breeding Int. Cambridge | NA | GN | late, short | Div |
| Florida | 1985 | Saatzucht Schweiger | NA | US | early, short | Div |
| INTRO 615 | NA | NA | NA | US | early, short | Div |
| Mironovskaya 808 | NA | Mironovska Research Institute | NA | UA | early, tall | Div |
| NS 22/92 | NA | Institute of Field and Vegetable Crops in Novi Sad, Serbia | NA | SR | early, tall | Div |
| NS 46/90 | NA | Institute of Field and Vegetable Crops in Novi Sad, Serbia | NA | SR | early, short | Div |
| NS 66/92 | NA | Institute of Field and Vegetable Crops in Novi Sad, Serbia | NA | SR | early, short | Div |
| Vel | NA | Purdue Univ., Indian | NA | US | early, tall | Div |

Table S2: Summary of the plant protection measures

|  | **Growth stage** | **Spraying agents** | | |
| --- | --- | --- | --- | --- |
|  |  | **2015** | **2016** | **2017** |
| **Herbicide** | **BBCH 21/25** | 1,2 l/ha Axial | 1,2 l/ha Axial | 1,2 l/ha Axial |
|  | **BBCH 25/29** | 70 g/ha Biathlon4D | 70 g/ha Biathlon4D | 70 g/ha Biathlon4D |
|  |  | 1 l/ha Dash | 1 l/ha Dash |  |
|  |  | 20 g/ha Dirigent SX | 20 g/ha Dirigent SX |  |
| **Fungicide** | **BBCH 31/37** | 2 l/ha Capalo | 2 l/ha Capalo | 1,6 l Capalo |
|  | **BBCH 49/55** | 1,1 l/ha Adexar | 1,1 l/ha Adexar | 2 l/ha Adexar |
|  |  | 1,1 l/ha Diamant | 1,1 l/ha Diamant |  |
|  | **BBCH59/61** | 1 l/ha Prosaro | 1 l/ha Prosaro | 1 l/ha Prosaro |
| **Growth regulator** | **BBCH 25/29** | 1,2 l/ha Stabilan 720 |  |  |
|  | **BBCH 31** | 0,5 l/ha Stabilan 720 | 0,5 l/ha Stabilan 720 | 1,5 l/ha Medax Top |
|  |  | 0,5 l/ha Medax Top | 0,5 l/ha Medax Top |  |
| **Insecticide** |  | 0,2 l/ha Sumicidin | 0,2 l/ha Sumicidin | 0,2 l/ha Sumicidin |
|  |  |  | 0,075 l/ha Karate Zeon |  |

Table S3: P-values from the ANOVA results for the yield and senescence parameters. No interaction effect for BBCH59 because of only one value per year, p-value for the factor year was calculated with cultivar as random factor. In all other calculations cultivar, year and the interaction were set as fixed in the linear mixed model.

|  | p-value for Factor effect | | |
| --- | --- | --- | --- |
|  | **Cultivar** | **Year** | **Interaction** |
| **Seed yield** | < 0.001 | < 0.01 | < 0.001 |
| **Biomass^[[1]](#footnote-1)^** | < 0.001 | < 0.01 | n.s. |
| **Harvest Index** | < 0.001 | < 0.01 | n.s. |
| **TKW** | < 0.001 | n.s. | < 0.001 |
| **Spikes per m²** | < 0.001 | < 0.01 | n.s. |
| **Grains per ear** | < 0.001 | < 0.05 | < 0.001 |
| **Grains per m²** | < 0.001 | n.s. | n.s. |
| **BBCH59** | < 0.001 | < 0.001 | - |
| **LAI_max_** | < 0.001 | < 0.05 | < 0.001 |
| **SPAD^[[2]](#footnote-2)^** | < 0.001 | n.s. | < 0.05 |
| **GLA_50_** | < 0.001 | < 0.01 | < 0.001 |
| **LAD** | < 0.001 | < 0.05 | < 0.001 |
| **GCD** | < 0.001 | < 0.05 | < 0.001 |

Table S4: Nitrogen content of the soil in [kg/ha] and amount of fertilized Nitrogen in the last row for every season. Three applications: at vegetation start, at stem elongation and around booting.

|  | **2015** | **2016** | **2017** |  |
| --- | --- | --- | --- | --- |
| **before sowing (0-60 cm depth)** | 149.9 | 18.1 | 22.8 |  |
| **at vegetation start (0-90 cm depth)^[[3]](#footnote-3)^** | 70.8 | 29.3 | 43.1 |  |
|  |  |  |  |  |
| **after harvest (0-90 cm depth)** | 86.6 | 34.2 | 28.9 |  |
|  | 150 | 190 | 180 | Fertilization in three applications |

Table S5: eather conditions during the experimental periods in comparison with the 30 years mean values from the DWD (DE's National Meteorological Service) for the relevant region.

|  | **Sowing date** | **Avg. heading date (BBCH 59)** | | | **Precipitation sum (March - July) [mm]** | **Avg. Temp. (Oct - July) [°C]** |
| --- | --- | --- | --- | --- | --- | --- |
|  |  | days after sowing | | Tsum [°Cd] |  |  |
| **2015** | 28^th^ Oct 2014 | 221 | | 1461 | 201.8 | 11.92 |
| **2016** | 27^th^ Oct 2015 | 217 | | 1618 | 235.7 | 12.73 |
| **2017** | 2^nd^ Nov 2016 | 213 | | 1436 | 325.7 | 13.42 |
| **30year mean from DWD** | | |  | | 296.3 | 11.6 |

Table S6: Broad-sense heritability (last column ”Overall”) and repeatability calculated within each year, Within each year the repeatability was calculated accordingly but without the cultivar – season interaction σ_CY_^2^ and the residual variance σ_e_^2^ divided by two instead.

|  | **2015** | **2016** | **2017** | **Overall** |
| --- | --- | --- | --- | --- |
| **Seed yield** | 0.78 | 0.68 | 0.76 | 0.74 |
| **Grains per spike** | 0.76 | 0.64 | 0.66 | 0.75 |
| **BBCH 59** | - | - | - | **0.87** |
| **LAI_max_** | 0.57 | 0.51 | 0.36 | 0.50 |
| **SPAD** | - | 0.65 | 0.56 | 0.66 |
| **LAD** | 0.71 | 0.59 | 0.62 | 0.51 |
| **GCD** | 0.75 | 0.75 | 0.79 | 0.57 |

1. Calculated from Harvest Index und final plot yield [↑](#footnote-ref-1)
2. only for 2016 and 2017 [↑](#footnote-ref-2)
3. measured in April, after the first fertilisation [↑](#footnote-ref-3)
